# Elevated high‐density lipoprotein levels following acute graft‐versus‐host disease onset: a potential link to T‐cell dysfunction and increased relapse risk

**DOI:** 10.1002/cti2.70060

**Published:** 2025-11-03

**Authors:** Romy Böttcher‐Loschinski, Franziska Karl, Diana Drettwan, Johannes Wittmann, Benedikt Jacobs, Simon Völkl, Heiko Bruns, Andreas Mackensen, Dimitrios Mougiakakos

**Affiliations:** ^1^ Department for Hematology, Oncology, and Cell Therapy Otto‐von‐Guericke University Magdeburg Germany; ^2^ Department of Medicine 5, Hematology and Oncology Friedrich‐Alexander‐Universität Erlangen‐Nürnberg and University Hospital Erlangen Erlangen Germany; ^3^ lifespin GmbH Regensburg Germany; ^4^ Health Campus Immunology, Infectiology, and Inflammation, Medical Center Otto‐von‐Guericke University Magdeburg Germany

**Keywords:** allo‐SCT, GvHD, GvL, HDL, relapse, T cells

## Abstract

**Objectives:**

Allogeneic stem cell transplantation (allo‐SCT) is the only curative treatment option for several haematologic malignancies. Its therapeutic principle is based on the donor T cells' ability to eliminate any residual malignant cells. Despite its success, challenges such as graft‐versus‐host disease (GvHD) and disease relapse persist. Recent studies emphasise the role of the metabolic environment in shaping T‐cell responses. This study investigates the impact of serum metabolites on T‐cell responses following allo‐SCT.

**Methods:**

Metabolite levels in serum samples from 55 allo‐SCT patients transplanted between November 2015 and October 2018 were analysed by nuclear magnetic resonance (NMR) spectroscopy for six time points after transplantation. These metabolite profiles were correlated with clinical data and T‐cell characteristics obtained by flow cytometry‐based immunomonitoring. High‐density lipoprotein (HDL) emerged as a key factor of interest. To explore the potential relationship between T‐cell‐related differences and HDL levels, healthy donor T‐cell cultures supplemented with HDL were performed.

**Results:**

Elevated HDL levels were associated with acute GvHD (aGvHD) and relapse. Patients with high HDL serum levels exhibited a delayed normalisation of T‐cell frequencies and increased effector‐memory CD8^+^ T‐cell frequencies. *In vitro* experiments revealed reduced proliferation and expression of activation/effector molecules after exposure to HDL. Effects of HDL on memory T‐cell subset formation resembled the *in situ* findings in patients.

**Conclusions:**

AGvHD was linked to elevated HDL levels, potentially affecting T‐cell‐mediated graft‐versus‐leukaemia (GvL) activity and promoting relapse. HDL could therefore be a potential biomarker for the success of allo‐SCT and a lever for improving patients' outcomes.

## Graphical abstract

Serum levels of high‐density lipoprotein (HDL) have been observed to increase upon the onset of acute graft‐versus‐host disease. In addition, elevated HDL has been shown to inhibit T cells, which could potentially compromise the graft‐versus‐leukaemia effect and elevate the risk of relapse. Figure was created with Biorender.com

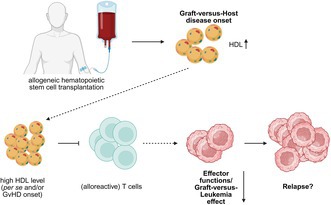

## Introduction

Disease relapse and graft‐versus‐host disease (GvHD) represent strong limitations for the success of allogeneic stem cell transplantation (allo‐SCT).^1^ Both, graft‐versus‐leukaemia/lymphoma (GvL) effect and GvHD are largely mediated by donor T cells. Therefore, fine‐tuning their effector functions is crucial for the success of allo‐SCT. The relationship between T‐cell metabolism and function, also known as immunometabolism,^2^ provides new possibilities for controlling T‐cell behaviour in cancer.

Both CD4^+^ and CD8^+^ T‐cell subsets contribute to GvHD and GvL. Allogeneic CD8^+^ T cells play an important role in the GvL reaction by targeting and eliminating remaining malignant cells. Yet, they also mediate GvHD.^3^, ^4^ CD4^+^ T cells primarily function as immune response facilitators, activating other immune cells involved in GvHD and GvL.^3^, ^5^ Additionally, CD4^+^ T cells can exhibit cytotoxicity against leukaemic blasts.^6^ T‐cell differentiation status also influences GvHD development. Activated naïve T cells (T_N_) make a significant contribution to GvHD induction, while effector memory T cells (T_EM_) and central memory T cells (T_CM_) have a minor role in GvHD induction but are critical for GvL responses.^3^, ^7^

T cells dynamically adjust their metabolism based on their differentiation status, microenvironmental conditions and challenges such as nutrient competition. For instance, highly glycolytic tumors compete for glucose with adjacent T cells. Consequently, T cells lack crucial intermediates from glycolysis necessary for biosynthetic processes, compromising their effector functions.^8^, ^9^ Excess glycolysis further leads to the abundant release of lactic acid, which inhibits T‐cell effector functions, proliferation and induces cell death.^10^, ^11^, ^12^ Lipids are other important determinants of T‐cell fate and function in a variety of physiological and pathological settings. Whereas short‐chain fatty acids, such as butyrate, enhance CD8^+^ T‐cell effector functions, an excess of exogenous fatty acids (FA) impairs T‐cell function and viability by increasing fatty acid oxidation (FAO), oxidative stress, mitochondrial damage, lipid peroxidation and inducing cell death.^13^, ^14^, ^15^, ^16^ High cholesterol levels in the tumor microenvironment lead to CD8^+^ T‐cell exhaustion.^17^

In contrast to the immunosuppressive microenvironment of tumor‐infiltrating T cells, alloreactive T cells face chronic stimulation, activation and an inflammatory environment after allo‐SCT.^18^ Following allo‐SCT, alloreactive T cells upregulate their metabolic activity, including FAO, oxidative phosphorylation (OXPHOS), glycolysis and the pentose phosphate pathway (PPP).^18^ The mTOR and AMPK signalling is activated to a greater extent in alloreactive T cells as compared to syngeneic T cells.^18^ Moreover, recent studies reported that similar to the observations from the tumor microenvironment, metabolites can affect reconstituting T cells after allo‐SCT. Acute myeloid leukaemia (AML) with high glycolytic activity abundantly releases lactic acid, which interferes with the T cells' bioenergetic activity and effector functions in relapsing allo‐SCT patients.^12^ Following allo‐SCT, accumulation of reactive oxygen species results in oxidative stress, which promotes oxidative DNA damage, exhaustion and functional aberrations in reconstituting T cells, thus leading to increased relapse risk.^19^

Therefore, we were interested in the systemic metabolic milieu, the corresponding T‐cell phenotype and the impact on the clinical course of allo‐SCT patients. To do so, we analysed the serum metabolites of 303 samples from six consecutive time points (Day +30, +45, +60, +70, +90, and +120 post allo‐SCT) of 55 allo‐SCT patients using nuclear magnetic resonance (NMR) spectrometry. Furthermore, we acquired clinical data and flow cytometry data regarding T‐cell status for those patients.

## Results

### Cluster analysis of metabolites in allo‐SCT patients

**Figure 1:**
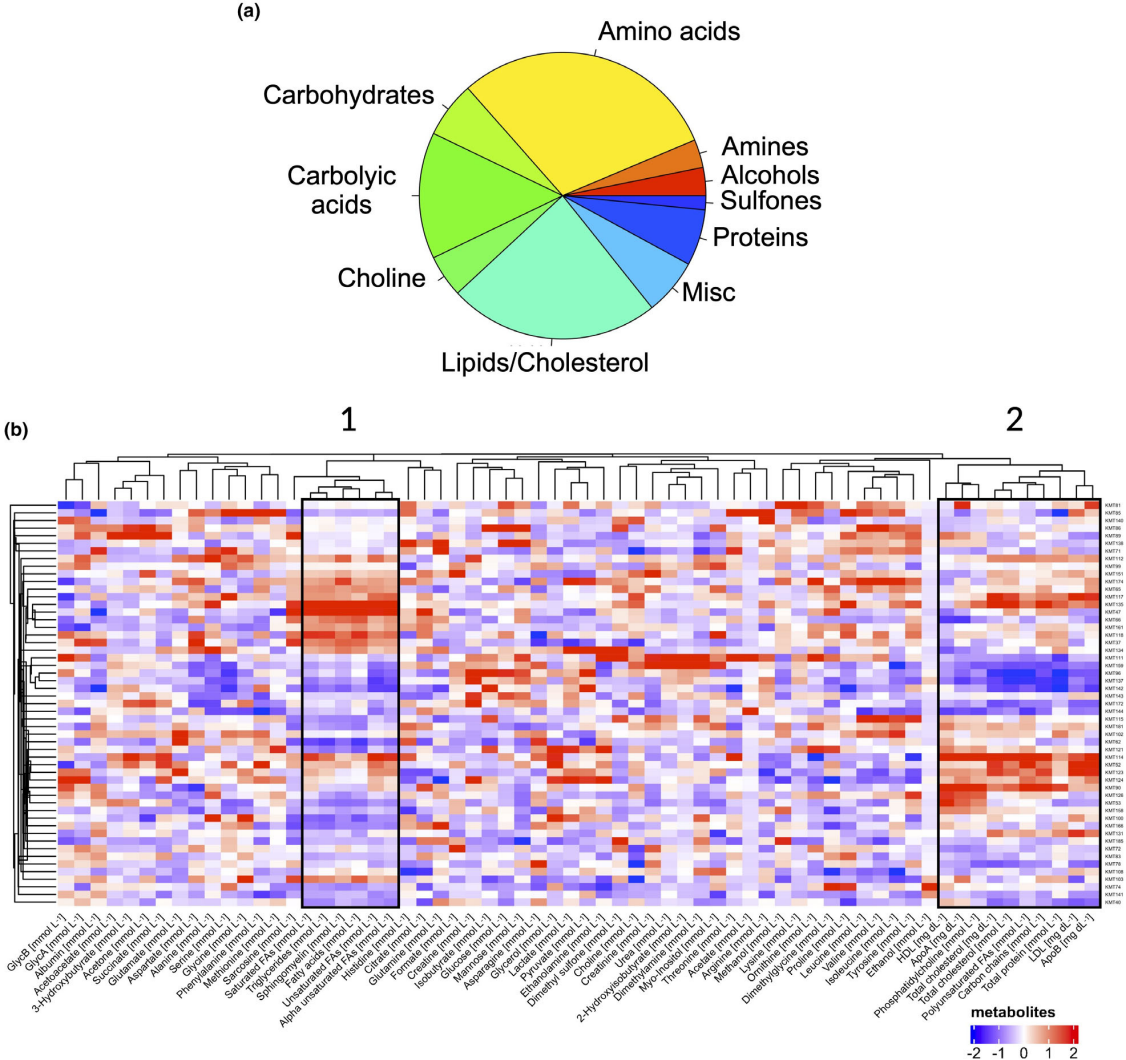
Analysis of metabolites in the serum of allo‐SCT patients using NMR spectroscopy. **(a)** Proportion of metabolite classes of all detected metabolites. **(b)** Heatmap of mean values of each metabolite for each patient. For comparison, data has been scaled across all patients and metabolites. After hierarchical clustering of metabolites, two clusters were identified based on serum levels of fatty acids, triglycerides and cholesterol.

The majority of the identified metabolites belonged to the categories of amino acids, lipids and carboxylic acids (Figure 1a). Concentrations of all metabolites remained relatively constant across all time points except for dimethyl sulfone and glycoprotein acetylation (data not shown). Thus, mean values were calculated for each metabolite and patient for hierarchical clustering. Patients were primarily clustered according to their FA and cholesterol profiles (Figure 1b). Cluster 1 consists of FAs, triglycerides and sphingomyelin, whereas Cluster 2 consists of phosphatidylcholine, cholesterol, HDL, LDL and the apolipoproteins A and B. Consistent with previous findings,^20^ patients with high cholesterol levels were inversely associated with low triglyceride/FA levels (Figure 1b). Additionally, more than half of the male patients had HDL cholesterol levels that were lower than the recommended levels of 40 mg dL^−1^.^21^ In contrast, female patients had higher levels of both total and HDL cholesterol as compared to male patients (Supplementary figure 1a). Because menopause can affect serum lipid levels,^22^ we compared female patients of different age groups with respect to serum triglyceride, total cholesterol, HDL and LDL levels. Given menopause usually begins between the ages of 45 and 55 years,^23^ female patients were divided into two groups: (1) ≤ 45 years and (2) > 45 years. Although there were no significant differences between these two groups, female patients older than 45 years had slightly higher triglyceride, total cholesterol and LDL levels, whereas HDL levels were slightly decreased (Supplementary figure 1b). Anti‐hormonal treatments were administered to all four female patients under the age of 45. Four of the female patients over 45 years of age also received anti‐hormonal treatments. No female patient received oestrogen supplements. There were no differences in the serum levels of triglycerides, cholesterol, HDL or LDL between patients who received anti‐hormonal treatment and those who did not (data not shown). Interestingly, the same trends in those metabolites were also observable within the male patient population (Supplementary figure 1c), indicating a rather age‐related than hormone‐dependent effect.

### Increased HDL levels are associated with aGvHD and relapse

**Figure 2:**
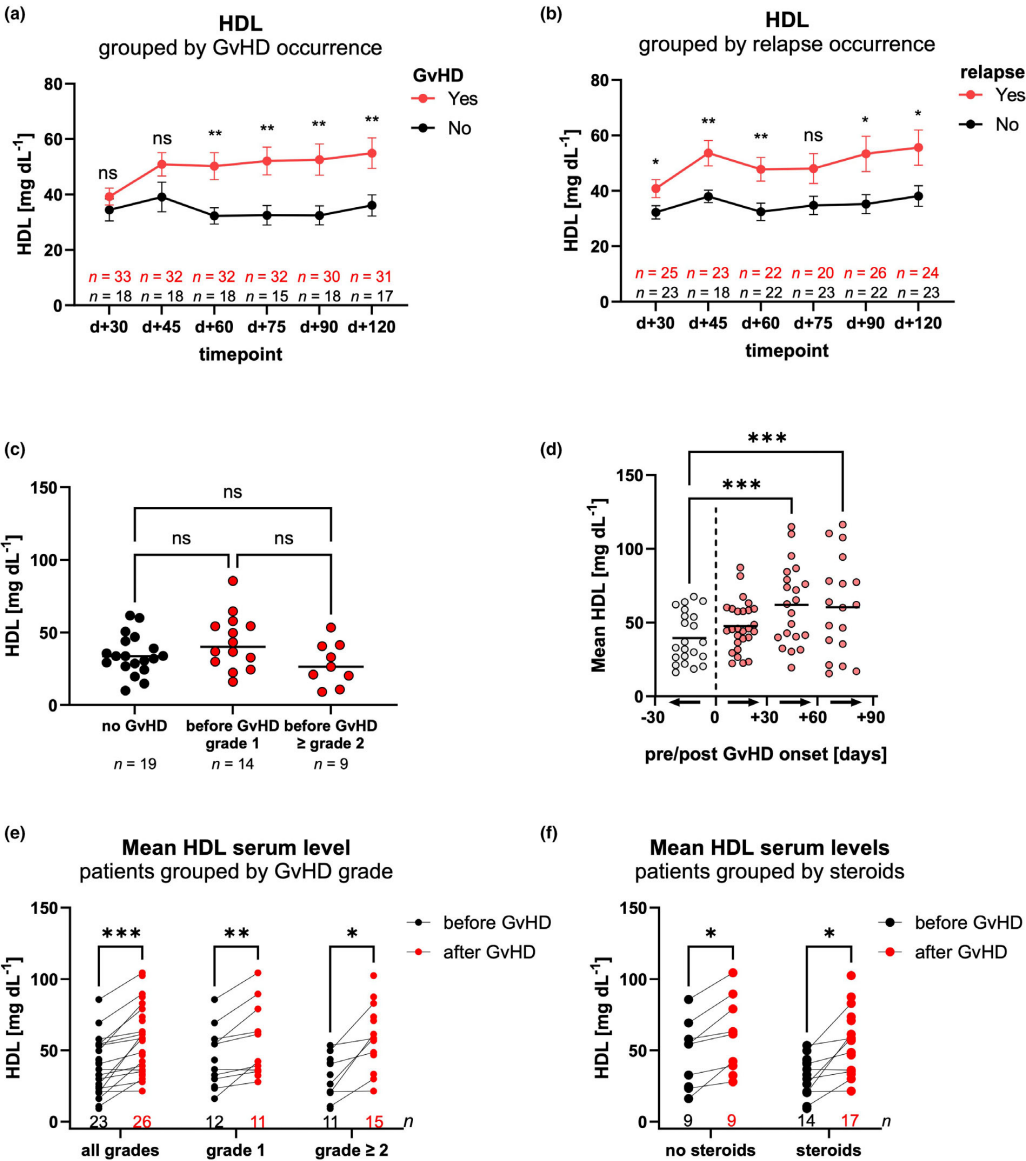
High HDL levels are associated with aGvHD and relapse after allo‐SCT. **(a, b)** Patients were grouped according to the occurrence of aGvHD **(a)** or relapse **(b)**. Changes in HDL serum levels over time are shown for these groups from Day +30 to Day +120 after allo‐SCT. **(c)** HDL serum levels are shown for three groups of patients: those who did not develop aGvHD, patients prior to the development of aGvHD grade 1 (no systemic steroids), and patients prior to the development of aGvHD grade ≥ 2. **(d)** HDL serum levels of allo‐SCT patients before and after GvHD onset were binned into 30‐day intervals from 30 days before to 90 days after GvHD onset and mean levels were calculated for these bins. **(e)** Mean HDL serum levels were calculated for allo‐SCT patients before and after GvHD onset and grouped based on their maximum GvHD grade. **(f)** Mean HDL serum levels were calculated for allo‐SCT patients before and after GvHD onset and grouped based on systemic corticosteroid treatment for GvHD. aGvHD, acute graft‐versus‐host disease; d, day; HDL, high‐density lipoprotein; LDL, low‐density lipoprotein; ns, not significant. Error bars indicate SEM. *P*‐values: *< 0.05, **< 0.01, ***< 0.001.

Next, we were interested in examining whether specific metabolites were associated with aGvHD and/or disease relapse. Interestingly, patients who developed aGvHD and/or relapse showed the highest HDL serum levels across all time points post‐transplant (Figure 2a and b). Notably, in the present cohort, 12 of 26 patients (46%) without aGvHD relapsed, compared with 18 of 34 patients (52%) who developed aGvHD. In contrast to HDL, LDL serum levels showed only minor differences between patients with aGvHD and without aGvHD. Triglyceride levels did not differ between the two groups (Supplementary figure 2a). Furthermore, LDL and triglyceride levels were not significantly different between patients who relapsed and patients who did not experience relapse (Supplementary figure 2b). To explore potential confounding factors influencing HDL levels, we analysed associations with smoking,^24^ diabetes^25^ and renal function.^26^ HDL levels did not differ between smokers and non‐smokers, nor between patients with diabetes requiring insulin treatment post‐transplant and non‐diabetic patients (Supplementary figure 2c). In addition, there was no correlation between HDL and creatinine levels (Supplementary figure 2d), suggesting that renal function, as assessed by creatinine, did not influence HDL levels in our cohort.

Because immunosuppressive drugs have also been reported to induce disturbances in serum lipid and cholesterol levels,^27^, ^28^, ^29^, ^30^ we compared serum fatty acid, triglyceride and cholesterol levels in relation to the different GvHD prophylaxis regimens (Supplementary figure 3a). We found increased fatty acid and triglyceride levels in patients receiving MMF/not receiving MTX compared to patients without MMF/with MTX. Total cholesterol, HDL and LDL serum were not statistically significant (Supplementary figure 3b). Furthermore, there were no significant differences between patients receiving CsA/ATG compared to patients receiving ptCY/Tac/MMF but no CsA (Supplementary figure 3c). In conclusion, HDL serum levels were not impacted by different GvHD prophylaxis strategies.

Additionally, treatment with lipid‐regulating agents could impact cholesterol and lipid serum levels in allo‐SCT patients. In our cohort, five out of 53 patients received statins prior to transplantation, which were discontinued for allo‐SCT. No other lipid‐lowering agents such as fibrates, ezetimibe or anion exchange resins were used before or after allo‐SCT. Prior statin treatment did not significantly alter serum lipid or cholesterol levels (Supplementary figure 3d).

The liver and the intestine are the primary producers of HDL.^31^ Moreover, these organs are typical sites of aGvHD. Therefore, we compared HDL serum levels in accordance with the organ(s) affected by aGvHD. The onset of liver and/or gastrointestinal aGvHD had no discernible effect on HDL levels compared to patients with GvHD who did not experience liver or gastrointestinal involvement. Interestingly, patients with skin involvement also exhibited significantly higher HDL levels than patients without GvHD (Supplementary figure 4a). The occurrence of liver toxicity (defined by serum bilirubin levels > 2 mg dL^−1^ at any point following allo‐SCT) did not result in significant differences in HDL serum levels (Supplementary figure 4b).

Since HDL levels are known to decrease during systemic infections,^32^ we investigated whether inflammation as well as viral (EBV, CMV), bacterial, or fungal infections affected HDL serum levels in our cohort. HDL serum levels did not correlate with the corresponding CRP values of the same time point (Supplementary figure 4c). During the observation period, 40 patients experienced EBV reactivation, 23 had CMV reactivation, 33 had confirmed bacterial infections, and 22 patients developed fungal infections. HDL serum levels did not differ significantly between patients with or without these events (Supplementary figure 4d), and no consistent changes were observed in HDL levels before versus after infection or reactivation events (Supplementary figure 4e).

In order to assess whether HDL levels were increased already before GvHD onset, we compared mean HDL serum levels of patients without GvHD with HDL levels of patients before getting GvHD grade 1 or GvHD grade 2 and higher (Figure 2c). Within the first 30 days, eight patients exhibited signs of graft‐versus‐host disease (GvHD). For the patients in question, no metabolite levels were assessed prior to the onset of GvHD. Consequently, these patients were excluded from Figure 2c. Out of the 26 other patients with GvHD, three patients did not have measurements before GvHD onset because of limited sample availability. HDL measurements from the 23 remaining patients (with one to five samples each) were averaged to yield a single pre‐onset value per patient. No significant differences were observed prior to GvHD onset compared to patients without GvHD. We therefore examined whether GvHD levels increased upon GvHD onset. Since the majority of data points covered a period between 30 days before and 90 days after GvHD onset, we limited our analysis to this time frame (Figure 2d). A significant rise in HDL was detected within the first 90 days following GvHD onset, compared to up to 30 days prior to onset. This increase after GvHD onset was independent of the maximal grade of aGvHD (Figure 2e), systemic treatment with corticosteroids (Figure 2f) and response to corticosteroid treatment (Supplementary figure 5a). In addition, the increase of HDL was observed in both males and females (Supplementary figure 5b). To assess the combined impact of clinical and transplant‐related parameters on HDL levels over time, we performed a linear mixed‐effects model including key transplant parameters (Supplementary figure 5c). The analysis revealed that the presence of GvHD was significantly associated with higher HDL levels (estimate = +14.54, *P* = 0.0056). There was a trend towards lower HDL levels in males (estimate = −12.82, *P* = 0.055), though age, donor match status, and days post‐transplant were not significantly associated with HDL. These results support the hypothesis that the onset of GvHD contributes to elevated HDL levels independently of time and baseline characteristics.

Consistently with the HDL increase after GvHD onset, serum HDL levels at the timepoint before aGvHD onset were not sufficient to predict aGvHD (Supplementary figure 5d). Prediction of relapse with serum HDL levels at the time point before relapse showed better performance but still did not achieve sufficient predictive accuracy for clinical implementation (Supplementary figure 5e), indicating that HDL alone is insufficient as a standalone biomarker. In summary, HDL levels increased in patients after aGvHD onset.

### T cell compartment in HDL^high^ and HDL^low^ allo‐SCT patients

**Figure 3:**
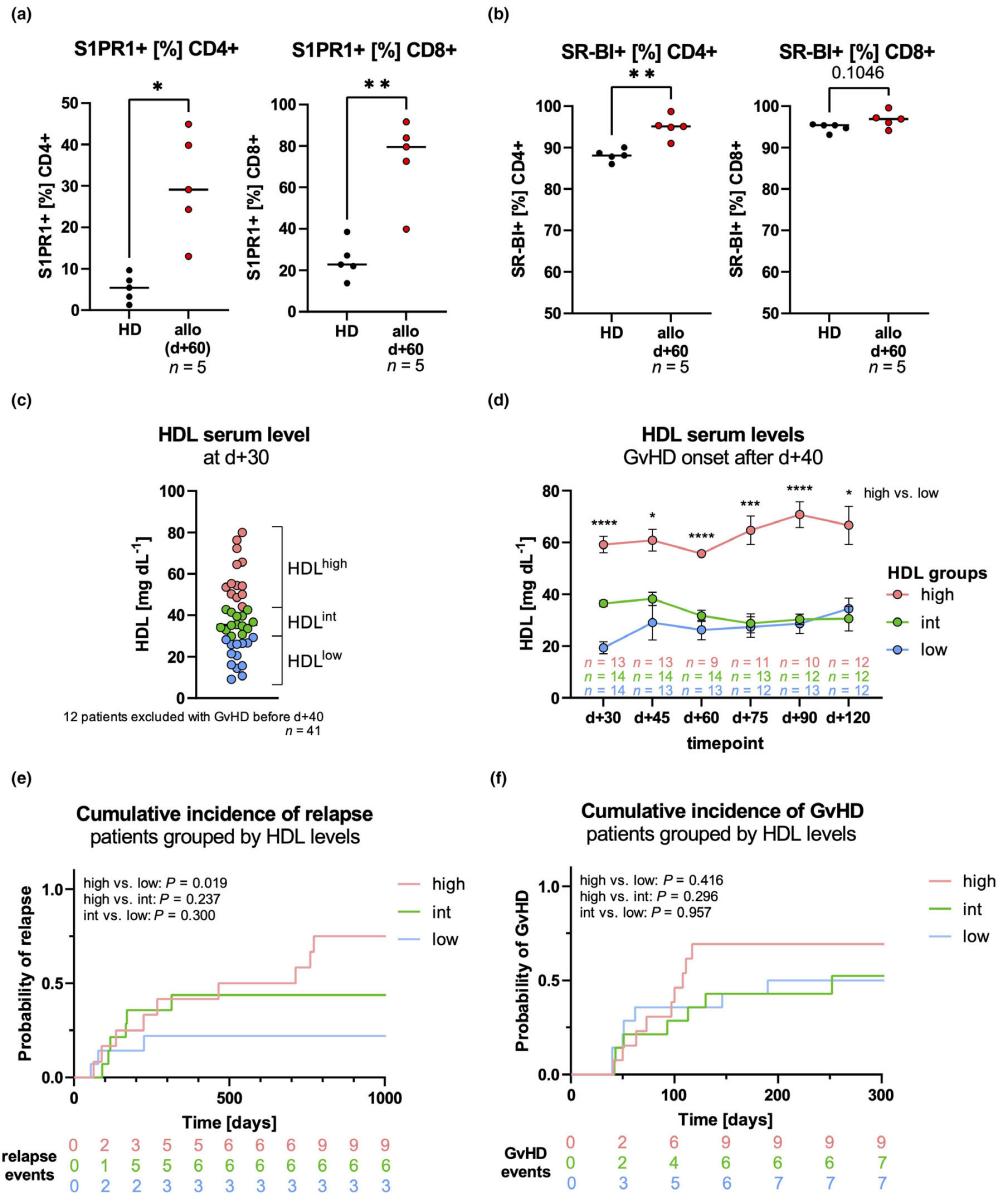
Classification of patients into HDL groups based on mean serum HDL levels. **(a, b)** The percentages of T cells that are positive for the HDL receptors S1PR1 **(a)** and SR‐BI **(b)** are shown for CD4^+^ and CD8^+^ T cells of HD and allo‐SCT patients at Day +60 post‐transplant (*n* = 5). **(c–f)** Patients were grouped according to their HDL serum levels at Day +30. Patients who already developed aGvHD around Day +40 were excluded from grouping and further analyses. **(c)** Dotplot of HDL serum levels at Day +30 indicating HDL patient groups. **(d)** The progression of serum HDL levels is shown for the respective HDL groups over the entire observation period of Day +30 until Day +120 post‐transplant. Significances are shown for the comparison of HDL^high^ vs. HDL^low^ serum levels of HDL for each timepoint. **(e, f)** Cumulative incidences of relapse and aGvHD are shown for the respective HDL extreme groups. aGvHD, acute graft‐versus‐host disease; allo, allo‐SCT patient; HD, healthy donor; HDL, high‐density lipoprotein; S1PR1, sphingosine‐1‐phosphate receptor 1; SR‐BI, scavenger receptor class B type 1. Error bars indicate standard error of mean. *P*‐values: *< 0.05, **< 0.01, ***< 0.001, ****< 0.0001.

It is well‐established that nutrient availability and metabolic signalling have a complex relationship with immune cell function.^33^ Therefore, we investigated whether elevated HDL levels might impact T‐cell reconstitution, differentiation, and function in allo‐SCT patients. Our analysis revealed that these patients had a higher proportion of sphingosine‐1‐phosphate receptor 1 (S1PR1)‐positive and scavenger receptor class B type 1 (SR‐BI)‐positive T cells compared to healthy donors (HD) (Figure 3a and b). Both S1PR1 and SR‐BI are known receptors for HDL. As shown in Figure 2b, serum HDL levels differ as early as Day 30 after transplantation between patients who relapsed and those who did not. We therefore hypothesised that high HDL levels already at baseline have a negative effect on T cells in allo‐SCT patients compared to lower HDL levels, possibly leading to relapse. Patients were divided into HDL groups based on their serum HDL levels at Day +30 post‐transplantation. In order to avoid possible bias in T‐cell composition because of ongoing GvHD, patients with GvHD onset up to Day +40 were excluded from this analysis. Despite the exclusion of these patients from the study, there remained statistically significant differences in HDL serum levels (Supplementary figure 6a). Given the modest cohort size, a tertile‐based stratification was used to focus on the most extreme HDL values and thereby maximise contrast and statistical power between groups. HDL groups were defined as follows: The top 33% of patients with the highest serum HDL levels were defined as the HDL^high^ group, the middle 33% of patients with intermediate serum HDL levels as the HDL^int^ group, and the bottom 33% of patients with the lowest serum HDL levels as the HDL^low^ group (Figure 3c, Table 1, Supplementary table 1). Notably, patients of the HDL^high^ group were predominantly female (Table 1). There were no significant differences in BMI or statin treatment before transplantation among the three groups (Table 1). HDL^high^ patients maintained the highest HDL serum levels throughout the observation period (Figure 3d). According to the prior observation, relapse was more common in patients of the HDL^high^ group (Figure 3e). The cumulative incidence of aGvHD was not significantly different between the HDL groups (Figure 3f). However, more aGvHD cases occurred in the HDL^high^ group (9/13 in HDL^high^ vs. 7/14 in HDL^low^). There were no significant differences in survival among the three HDL groups (Supplementary figure 6b, Table 1). In order to detect a potential impact of HDL on patients' T cells, flow cytometry data of the reconstituting T cells^19^, ^34^ were compared between the two extreme groups HDL^high^ and HDL^low^ (Figure 3c).

**Table 1:** Descriptive statistics of HDL extreme groups

Variable	High (*n* = 13)	Int (*n* = 14)	Low (*n* = 14)	*P* overall
Sex				
Female	11 (84.6%)	3 (21.4%)	6 (42.9%)	0.004
Male	2 (15.4%)	11 (78.6%)	8 (57.1%)	
Age at Tx (years)	57.0 [20.0; 66.0]	58.0 [20.0; 72.0]	59.5 [19.0; 70.0]	0.57
BMI	25.5 [19.2; 55.8]	27.7 [22.4; 44.4]	26.8 [21.0; 32.1]	0.952
Time until GvHD onset (days)	97.0 [41.0; 117]	93.0 [42.0; 252]	51.0 [40.0; 190]	0.679
Survival time (days)	728 [698; 1098]	287 [35.0; 1056]	634 [129; 1317]	0.086
Patient died?				
No	8 (61.5%)	7 (50.0%)	10 (71.4%)	0.508
Yes	5 (38.5%)	7 (50.0%)	4 (28.6%)	
Relapse‐related death				
No	0 (0.00%)	1 (16.7%)	1 (25.0%)	0.714
Yes	5 (100%)	5 (83.3%)	3 (75.0%)	
Time until relapse onset (days)	366 [63.0; 1486]	142 [91.0; 314]	78.0 [54.0; 225]	0.125
Statins prior to Tx				
No	12 (92.3%)	14 (100%)	11 (78.6%)	0.199
Yes	1 (7.7%)	0 (0.00%)	3 (21.4%)	

BMI, body mass index; GvHD, graft‐versus‐host disease; Tx, transplantation.

**Figure 4:**
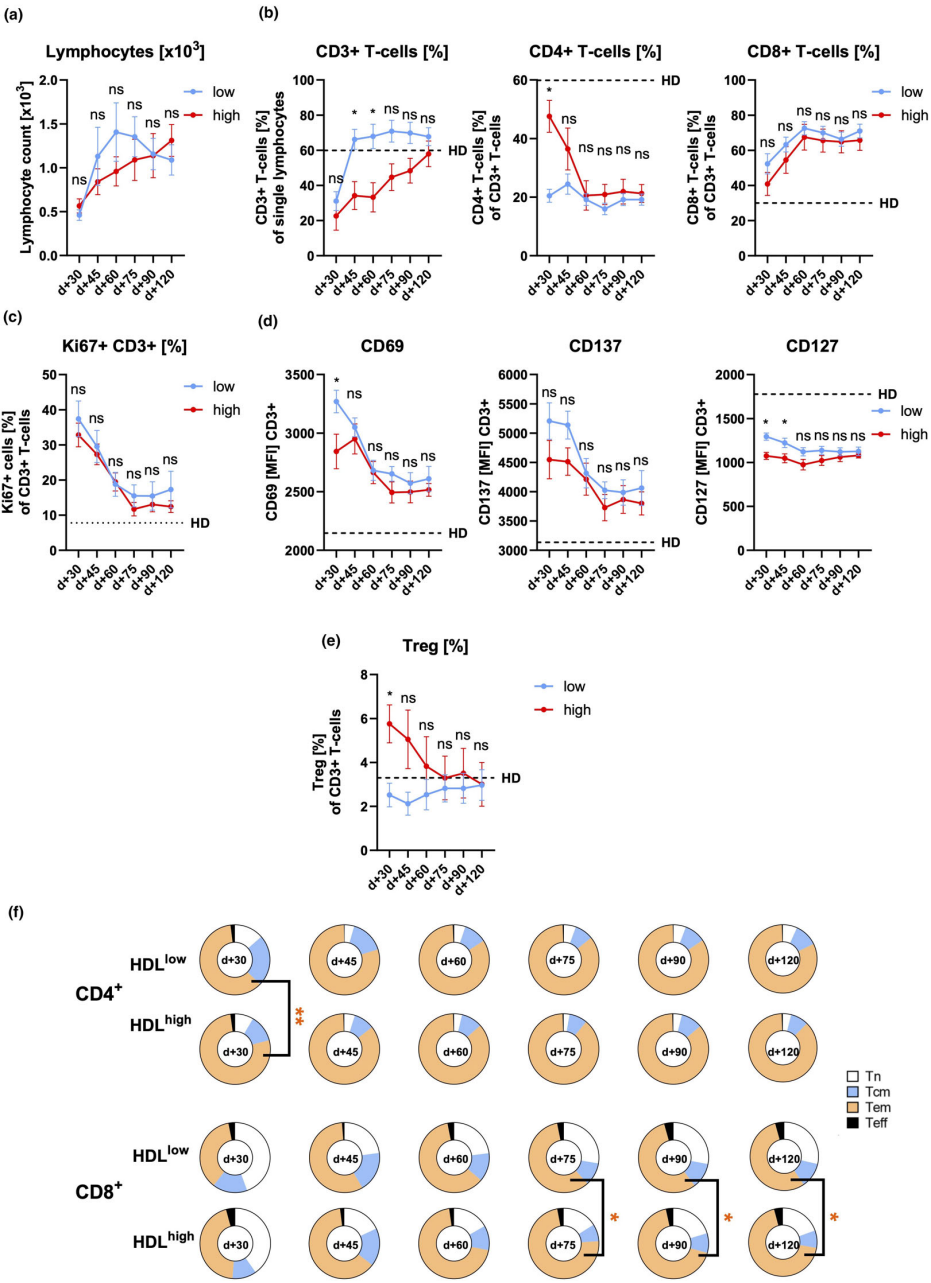
Immunomonitoring analysis of HDL extreme groups. **(a)** The absolute lymphocyte count, **(b)** frequencies of total, CD4^+^ and CD8^+^ T cells and **(c)** Ki67^+^ T cells, **(d)** MFIs of CD69, CD137 and CD127 of total T cells as well as **(e)** frequencies of Tregs are shown for the entire observation period (*n* = 9–14 per group and time point). **(f)** The frequencies of the respective differentiation states of CD4^+^ and CD8^+^ T cells (T_N_, T_CM_, T_EM_ and T_EFF_) are shown for each sampling time point following allo‐SCT (*n* = 9–14 per group and time point). T cell subsets were defined as: T_N_ CD45RO^−^ CCR7^+^, T_CM_ CD45RO^+^ CCR7^+^, T_EM_ CD45RO^+^ CCR7^−^, T_EFF_ CD45RO^−^ CCR7^−^. MFI, median fluorescence intensity; T_CM_, central‐memory T cell; T_EFF_, effector T cell; T_EM_, effector‐memory T cell; T_N_, naïve T cell; Treg, regulatory T cells. Error bars indicate standard error of mean. *P*‐values: *< 0.05, **< 0.01.

The reconstitution of the absolute lymphocyte count did not show a statistically significant difference between HDL^low^ and HDL^high^ patients. However, reconstitution appeared to occur faster in the HDL^low^ group (Figure 4a). Furthermore, besides CR status at transplantation and EBV reactivation, T‐cell reconstitution was also found to be significantly and independently affected by HDL levels in multivariate statistical analysis (Supplementary table 2, Supplementary figure 6c). Here, T‐cell reconstitution was defined as reaching 60% CD3^+^ T cells of live lymphocytes, which we found to be the mean T‐cell frequency in our HD cohort of the flow cytometric monitoring.

We further found that HDL^high^ patients had lower total T‐cell frequencies than HDL^low^ patients did early after allo‐SCT, with numbers converging during our follow‐up period (Figure 4b). The CD4/CD8 ratio initially shifted towards increased CD4^+^ T‐cell frequencies in the HDL^high^ group compared to the HDL^low^ group but equalised over time (Figure 4b). The proportion of proliferating Ki‐67^+^ T cells did not differ between the two extreme HDL groups (Figure 4c).

The expression of the activation markers CD69, CD137 and the naïve/memory marker CD127 on total CD3^+^ T cells was reduced in HDL^high^ patients 30 days after allo‐SCT, although the reduction was not significant for CD137. These expression levels also converged over time (Figure 4d). Additionally, frequencies of CD127^low^ CD25^high^ regulatory T cells were increased in patients with high HDL serum levels at Day +30 following allo‐SCT but declined thereafter resulting in no significant differences compared to HDL^low^ patients (Figure 4e). Given the established role of T‐cell metabolism in regulating T‐cell activation, function, and differentiation,^2^ the expression levels of GLUT1, HK2 and CPT1a were evaluated as part of the monitoring panel. Nevertheless, the expression levels of the aforementioned markers did not demonstrate any significant differences between the groups (Supplementary figure 6d).

CD4^+^ T‐cell differentiation differed only at Day +30 post transplantation between the two extreme groups (Figure 4f). At this time point, HDL^high^ patients had higher frequencies of T_EM_ cells and lower frequencies of T_CM_ cells. However, these differences started to disappear at Day +45 and were no longer detectable at Day +60. In contrast, CD8^+^ T‐cell differentiation initially followed a similar pattern but gradually diverged over time, resulting in higher T_EM_ frequencies as well as lower T_N_ and T_CM_ frequencies in HDL^high^ compared to HDL^low^ patients (Figure 4f). Because factors such as immunosuppression, infections, ongoing GvHD and others could affect CD8^+^ differentiation, we tested the impact of several factors on CD8^+^ T_EM_ frequencies. CD8^+^ T_EM_ frequencies increased significantly with days post‐transplant and CMV reactivation, which is also known as CMV‐related ‘memory inflation’.^35^ Importantly, also higher HDL levels were significantly associated with higher CD8^+^ T_EM_ frequencies. No other clinical or demographic factors exhibited an independent impact (Supplementary figure 6e).

In conclusion, patients with high levels of serum HDL display an altered T‐cell reconstitution and CD8^+^ T‐cell differentiation compared to patients with low HDL serum levels.

### HDL affects T‐cell activation, differentiation and effector functions *in vitro*

**Figure 5:**
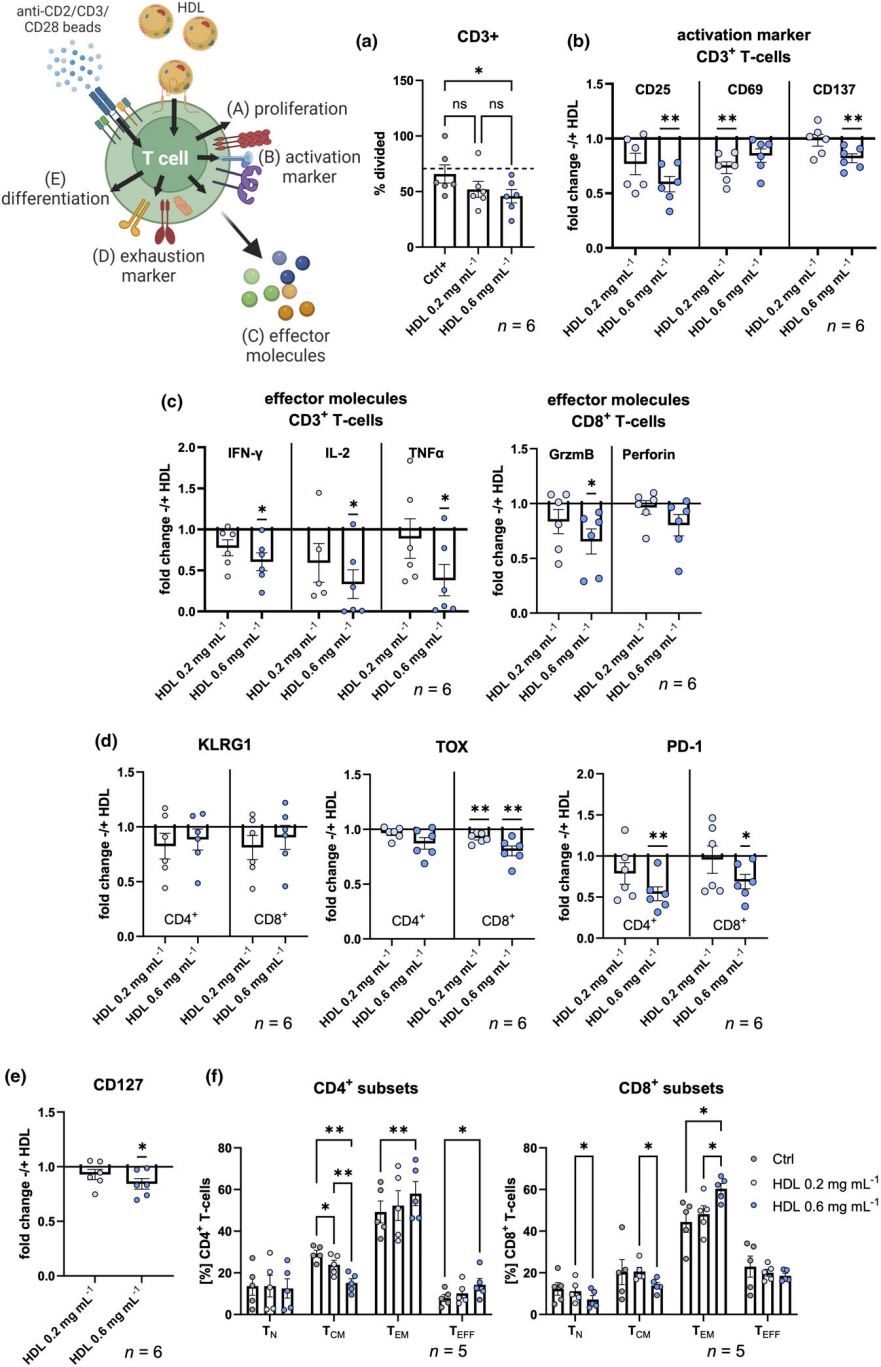
Effects of *ex vitro* short‐term HDL treatment of T cells. HD T cells were activated using anti‐CD2/CD3/CD28 activation beads with the indicated HDL concentrations or without HDL (= ctrl) for 120 h. **(a)** Proliferation was evaluated using flow cytometry, and the percentages of divided cells are presented. The dashed line represents the average percentage of divided cells observed in the activated control group (Ctrl+). Ctrl− represents the control group without incubation with activation beads. Expression of **(b)** activation markers, **(c)** effector molecules and **(d)** exhaustion markers are shown as fold change of median fluorescence intensity (MFI) compared to activated control cells without HDL. **(e)** Expression of CD127 is shown as fold change of MFI compared to activated control without HDL. **(f)** The differentiation subsets T_N_, T_CM_, T_EM_ and T_EFF_ are displayed for CD4^+^ and CD8^+^ T cells. The dashed lines indicate the average frequencies of the activated control without HDL. T cell subsets were defined as: T_N_ CD45RO^−^ CCR7^+^, T_CM_ CD45RO^+^ CCR7^+^, T_EM_ CD45RO^+^ CCR7^−^, T_EFF_ CD45RO^−^ CCR7^−^. HD, healthy donor; HDL, high‐density lipoprotein; MFI, median fluorescence intensity; T_CM_, central‐memory T cell; T_EFF_, effector T cell; T_EM_, effector‐memory T cell; T_N_, naïve T cell. Error bars indicate standard error of mean. *P*‐values: *< 0.05, **< 0.01. The overview figure was created with Biorender.com.

To further investigate the direct effect of HDL cholesterol on T cells, we treated healthy donor T cells *in vitro* with two different concentrations of HDL: 0.2 mg mL^−1^, which mimics the mean serum concentrations measured in HDL^low^ patients, and 0.6 mg mL^−1^, which mimics the mean serum concentrations in HDL^high^ patients. Both HDL concentrations slightly reduced the viability of T cells compared to the activated control. However, this effect was not significant (Supplementary figure 7a). The frequency of divided T cells was diminished in T‐cell cultures treated with the highest concentration of HDL (Figure 5a). It is noteworthy that when the analysis was restricted to proliferating cells, no significant difference in the number of divisions was observed between the control and HDL‐treated conditions (Supplementary figure 7b). These findings suggest that HDL inhibits proliferation in a subset of T cells but does not alter the proliferative capacity of those cells that do divide. The effects of HDL treatment on both CD4^+^ and CD8^+^ T‐cell subsets were also investigated. In CD8^+^ T cells, even low concentrations of HDL were sufficient to reduce proliferation, whereas CD4^+^ T cells required higher levels of HDL to exhibit similar effects (Supplementary figure 7c). Correspondingly, we observed significantly lower expression of the activation markers CD25, CD69 and CD137 (Figure 5b). The expression of effector molecules, including IFN‐γ, IL‐2, TNFα and granzyme B (GzmB), was reduced in CD8^+^ T cells when exposed to the highest concentration of HDL. However, while perforin expression exhibited a downward trend, it did not attain statistical significance (Figure 5c). CD4^+^ T‐cell differentiation into TH1, TH2 and TH17 was not altered by HDL treatment (Supplementary figure 7d). Furthermore, expression of the exhaustion/senescence marker KLRG1 was not significantly altered by HDL, whereas two other exhaustion/senescence markers, TOX and PD‐1, were found to be downregulated in a concentration‐dependent manner, particularly in CD8^+^ T cells (Figure 5d). Expression of CD127, the receptor for IL‐7, which is important for T_N_ differentiation and survival as well as memory T‐cell development and homeostasis,^36^ was also downregulated in T cells (Figure 5e). With regard to CD8^+^ T‐cell differentiation (Figure 5f), incubation with the higher concentration of HDL resulted in an increased frequency of T_EM_ cells and a decrease in the T_N_ and T_CM_ subsets as observed in the *ex vivo* analysis (Figure 4e). Interestingly, we also observed differences within the CD4^+^ T‐cell population in our *in vitro* cell culture, with increased frequencies of T_EM_ and effector T cells (T_EFF_) and decreased frequencies of T_CM_ cells (Figure 5f). Notably, as seen in the CD8^+^ T‐cell compartment, the effect on differentiation was only seen with the higher concentration of HDL, whereas differentiation with the lower concentration of HDL was similar to the control (Figure 5f).

## Discussion

GvHD and relapse continue to pose clinical challenges in allo‐SCT. T cells play a crucial role in both GvL and GvHD, and their adaptation to their metabolic milieu controls their function.^12^, ^37^ Therefore, we aimed to investigate the impact of circulating metabolites on the outcome of allo‐SCT and patients' T cells. In summary, aGvHD and relapse were associated with higher HDL serum levels. Furthermore, HDL levels increased independently of GvHD prophylaxis, GvHD treatment and infections. *Per se* higher HDL levels were associated with a certain pattern of T cell differentiation in allo‐SCT patients. Overall CD4^+^ T‐cell differentiation showed no significant differences between HDL extreme groups, except HDL^high^ patients initially had higher T_EM_ and lower T_CM_ frequencies at Day +30 compared to HDL^low^ patients. CD8^+^ T‐cell differentiation diverged over time, resulting in higher T_EM_ frequencies in HDL^high^ patients in comparison with HDL^low^ patients. *In vitro* treatment of HD T cells with high HDL concentrations affected T‐cell function in a way that, if also true for the allo‐SCT patient situation, could potentially hamper GvL activity and thereby promote relapse.

The majority of our patients displayed increased serum levels of triglycerides and/or cholesterol compared to guideline recommendations.^38^, ^39^ Hypercholesterolemia and hypertriglyceridemia after allo‐SCT have been previously reported.^40^, ^41^ Both can have various causes, such as obesity, GvHD grade and the use of immunosuppressants for GvHD prophylaxis/treatment.^27^, ^41^, ^42^ However, we found that neither BMI nor GvHD grade nor immunosuppression by CsA, MMF, ptCy, MTX or systemic corticosteroids showed an impact on HDL levels in our patient cohort.

Consistent with prior findings, male patients presented lower serum cholesterol levels compared to female recipients,^41^ which is discussed to be because of hormonal differences.^22^ Therefore, we examined cholesterol and lipid levels in pre‐ and peri‐/postmenopausal patients defined by age. Although triglycerides, total cholesterol and LDL increased with age—and HDL declined—these age‐related trends did not differ between the age groups and between male and female recipients. Importantly, increased HDL levels after aGvHD onset were further independent of sex suggesting a hormonal‐independent mechanism. Nevertheless, long‐term hormonal effects on serum lipid and cholesterol levels cannot be excluded. Interestingly, the usage of lipid‐lowering medication, especially statins, after allo‐SCT in cases of dyslipidaemia is already discussed in the literature with some reporting lower rates of clinically severe acute and/or chronic GvHD when given to donors or recipients.^43^, ^44^, ^45^ However, prospective and randomised data are limited; results are heterogeneous and even adverse effects, such as decreased GvL effects are reported.^43^ Other lipid‐targeting strategies, such as fibrates, niacin and PCSK9 inhibitors, have attracted interest because of their effects on lipoprotein composition and on innate/adaptive immune pathways, but remain experimentally and clinically understudied in the allo‐HCT setting.^46^

Given the inflammatory nature of aGvHD, increased HDL serum levels after GvHD onset were a rather unexpected finding, as HDL is largely considered to be anti‐inflammatory.^47^, ^48^ In fact, elevated HDL levels might act as a compensatory mechanism to increased inflammation during aGvHD. One study showed that infusing human HDL particles into murine preclinical aGvHD models acted protectively by eliminating circulating LPS.^49^ Furthermore, HDL mediates anti‐inflammatory responses in immune cells, such as the downregulation of toll‐like receptor‐mediated cytokine expression in macrophages.^50^ However, these increased HDL levels may also be a disadvantage as research on atherosclerosis has shown that HDL may lose its atheroprotective and anti‐inflammatory effects under certain circumstances, such as inflammation and oxidative stress, which are also present in GvHD.^19^ In these cases, HDL may even have pro‐inflammatory effects on the immune system, which could further promote GvHD.^51^, ^52^

Interestingly, HDL levels were found to increase independently of liver and gastrointestinal aGvHD, despite both being primary producers of HDL^31^ and common sites of GvHD. Elevated HDL levels were also found in patients experiencing skin aGvHD. This is consistent with reports showing that abnormal levels, composition and functionality of HDL particles also play a role in several skin diseases, such as the inflammatory atopic dermatitis and psoriasis.^53^ Consequently, the mechanism behind the observed increase in HDL levels remains unclear, but correlates with the onset of GvHD.

Nonetheless, prolonged exposure of T cells to high levels of HDL, not only after GvHD onset but also consistently after transplantation, could potentially affect T‐cell function and consequently the risk of relapse. Indeed, we could show that T cells from allo‐SCT patients express higher levels of HDL receptors S1PR1 and SR‐BI than HD T cells. In fact, it has been shown that T cells express HDL receptors, such as sphingomyelin receptors S1PRs and SR‐BI, and are modulated by HDL.^47^, ^54^, ^55^ Our observations show that CD3^+^, CD4^+^ and regulatory T‐cell frequencies along with CD69, CD137 and CD127 expressions show only transient differences between HDL^high^ and HDL^low^ patients directly after allo‐SCT. The relevance of these early changes for relapse remains unclear.

However, patients with high HDL serum levels showed a significant delay with time to T‐cell frequency normalisation, which was independent of remission status before transplantation and EBV reactivation. Whereas the HDL^low^ group and EBV reactivation were associated with shorter time to frequency normalisation, patients with a complete remission (CR) before allo‐SCT showed prolonged time. Patients in CR may have received more intensive prior therapy or different conditioning regimens that delay haematopoietic and immune reconstitution; alternatively, lower residual antigenic stimulation in CR patients could reduce homeostatic expansion of T cells and therefore prolong the time required to reach population norms. In our cohort, EBV, but not CMV reactivation showed significant differences in T‐cell frequency normalisation in univariate analysis, which might be because of different treatment strategies. Depletion of (EBV‐positive) B‐cells using rituximab as well as reduction in immunosuppression could increase the frequency of T cells in the blood, whereas CMV treatment directly targets the virus using antiviral drugs instead of cellular blood components. Elevated HDL may alter T‐cell reconstitution through effects on lymphocyte activation and proliferation, which we also detected in our *in vitro* assays with high HDL medium levels. HDL has previously been shown to inhibit T‐cell proliferation.^56^ Larbi *et al*.^57^ did show that proliferation is negatively affected by HDL from elderly donors, but not by HDL from younger donors. Furthermore, they demonstrated that HDL particles modulate lipid rafts by induction of cholesterol efflux, consequently affecting T cell signalling and HDL from elderly donors led to a decreased cholesterol efflux and cell membrane fluidity resulting in reduced proliferation. The reduced expression of CD69, CD137 and CD127 may also be linked to higher HDL levels, as seen in our *in vitro* experiments. In contrast, consistent differences were observed in CD8^+^ T‐cell differentiation, with an increase in T_EM_ and a decrease in T_N_ and T_CM_ frequencies, which were also replicated *in vitro*. Minimal differences in memory development of CD4^+^ T cells were observed *ex vivo* between HDL^high^ and HDL^low^ groups. However, our *in vitro* cultures under HDL^high^ conditions showed increased frequencies of CD4^+^ T_EM_ and T_EFF_ T cells.

The role of T_N_, T_CM_, and T_EM_ cells in the context of aGvHD/relapse remains controversial, with most studies performed in preclinical models. T_CM_ cells are linked to GvL responses, though their role in inducing aGvHD is unclear.^58^, ^59^ T_EM_ cells have been shown to have a reduced ability to induce aGvHD, but still maintain GvL activity.^59^ However, elevated CD8^+^ T_EM_ counts in allo‐SCT patients were associated with a higher aGvHD incidence,^60^ which is consistent with our observations in the HDL^high^ patient group. Nevertheless, the relationship between T cell populations and aGvHD/relapse is not fully understood and requires further studies.

In addition to differences in T‐cell differentiation and expression of CD69 and CD127, we observed decreased T‐cell proliferation and reduced levels of the activation marker CD25 and the exhaustion marker PD‐1 in our *in vitro* model. Furthermore, effector molecules such as IFN‐γ, IL‐2 and GzmB were lower expressed in CD8^+^ T cells. These effects were primarily associated with high HDL levels, supporting our hypothesis of a direct effect of high HDL levels on T‐cell activity. So far, reports of the impact of HDL on T‐cell cytokine production have shown mixed results. For example, an increased IL‐2 expression has been observed in T cells upon HDL treatment, which was decreased in our *in vitro* system.^57^ In contrast, other studies have reported that ApoA‐I and ApoA‐II proteins, major components of HDL particles, suppress the differentiation into pro‐inflammatory CD4^+^ T‐cell subsets and production of IFN‐γ.^61^, ^62^, ^63^ It is likely that differences in HDL function, concentration and lipid composition will result in different effects and should be explored further. In fact, HDL concentrations used in the literature for *in vitro* experiments vary widely, ranging from 0.05 to 0.6 mg mL^−1^.^56^, ^57^, ^64^ In our study, HDL concentrations corresponding to the HDL^low^ and HDL^high^ groups were used. The use of these concentrations demonstrated that high HDL concentrations inhibit T‐cell activation, proliferation and functionality and may contribute to relapse.

Although our study provided valuable insights, its limitations must be acknowledged. These limitations include a lack of information on the serum lipid levels and physical constitution of stem cell donors, which could clarify the link between donor characteristics and effects in allo‐SCT patients. The number of patients included was limited, and there is an imbalance in the manifestation and grades of GvHD. The analysis of HDL extreme groups and T‐cell characteristics is descriptive only and does not account for potential confounders such as immunosuppressive regimen duration. In addition, our short‐term *in vitro* experiments created a disparity in exposure time to HDL when compared to patients. Addressing these limitations in future studies will improve our understanding of the complexities associated with allo‐SCT and its impact on patient outcomes.

In summary, upon aGvHD onset an increase in circulating HDL levels was observed. *Per se* elevated HDL levels were associated with differences in T‐cell reconstitution and CD8^+^ T‐cell differentiation in those allo‐SCT patients. Furthermore, high HDL levels can inhibit T‐cell proliferation and function *in vitro*, which, if the same is true in the allo‐SCT setting, and/or in combination with increasing levels after GvHD onset, could potentially lead to alterations in the GvL effect and an increased risk of relapse. However, causality cannot be inferred from these data, and further validation in independent cohorts alongside mechanistic studies is required.

## Methods

### Patient samples

In accordance with the Declaration of Helsinki, peripheral blood samples were retrieved from patients for up to six different time points (Day +30, +45, +60, +70, +90, and +120) post allo‐SCT and HD upon informed consent (approval number of local ethic committees: 200_12, 280_14 B, 313_17B and 61/22). Patients transplanted between November 2015 and October 2018 were included in this study. Serum was collected and peripheral blood mononuclear cells (PBMCs) were isolated using Ficoll‐Paque (GE Healthcare, Chicago, IL, USA). The severity of GvHD at the time of diagnosis was determined using the MAGIC criteria.^65^ Patients' characteristics are summarised in Table 2 and Supplementary table 3.

**Table 2:** allo‐SCT patient characteristics

Variable	Patients (*n* = 53)
Sex	
Male	23 (43.4%)
Female	30 (56.6%)
Age (years)	57.0 [19.0; 72.0]
BMI	27.7 [19.2; 55.8]
Diagnosis	
AML/MDS	28 (52.8%)
Other	25 (47.2%)
Remission status at alloSCT	
CR	19 (35.8%)
PR/SD	34 (64.3%)
Donor type	
Haploidentical family member	6 (11.3%)
HLA‐identical sibling	8 (15.1%)
Matched unrelated donor	39 (73.6%)
GvHD prophylaxis	
CSA/MMF	26 (49.1%)
CSA/MTX	21 (39.6%)
Cyclophosphamide/tacrolimus/MMF	6 (11.3%)
Conditioning	
Myeloablative	24 (45.3%)
Non‐myeloablative	29 (54.7%)
Relapse	26 (48.1%)
aGvHD	34 (64.2%)
Maximal overall aGvHD grade	
1	15 (44.1%)
2	13 (38.2%)
3	3 (8.82%)
4	3 (8.82%)
Steroid treatment	24 (45.3%)

aGvHD, acute graft‐versus‐host disease; AML, acute myeloid leukaemia; CR, complete remission; CSA, cyclosporine A; MDS, myelodysplastic syndrome; MMF, mycophenolate mofetil; MTX, methotrexate; PR, partial remission; SD, stable disease.

### NMR spectroscopy of serum samples

#### Preparation of serum samples

Metabolite levels in serum samples from 55 allo‐SCT patients were assessed by NMR spectroscopy (lifespin GmbH, Regensburg, Germany). Once defrosted, samples are prepared by using 350 μL serum sample. To each sample, 350 μL of an aqueous buffer solution are added. The buffer contains p.A. quality H_2_O, 0.1 g L^−1^ NaN_3_, 0.067 mol L^−1^ Na_2_HPO_4_, 0.033 mol L^−1^ NaH_2_PO_4_ (pH‐value: 7.15 ± 0.05) and 5% D_2_O as a field locking substance. As an internal standard, 6 mM pyrazine are added. From this final solution, 600 μL are transferred to 5 mm Bruker NMR tubes and closed with barcode caps. The samples are stored at 4°C until subsequent NMR acquisition, which takes place within 24 h of sample preparation.

#### NMR measurement

NMR spectra are acquired on a 600 MHz Bruker Avance NEO NMR spectrometer equipped with a 5 mm BBI probe (Bruker, Billerica, MA, USA). 1D NMR spectra are recorded using a NOESY‐presaturation pulse sequence (noesygppr1d) with a spectral width of 30 ppm and 98 304 data points. The number of scans was set to 16, relaxation delays to 10 s and temperatures to 310 K for serum samples.

#### Data analysis

The obtained spectra of serum samples are Fourier transformed with TopSpin software version 4.1 (Bruker). All ^1^H‐NMR spectra are automatically phased and baseline corrected. Proprietary Lifespin Profiler software for Blood Biomarker Analysis is used for automated detection and quantification of serum/plasma metabolites. In total, 303 samples were assessed. One patient had to be excluded from further analysis because of methodological reasons. A second patient was excluded because of loss to follow‐up. Sample quantities included in this study are listed in Supplementary table 4. Metabolites that were not detected in more than 2/3 of the samples were excluded from further analysis. Consequently, 97 out of 121 metabolites were included in this study.

### Flow cytometry

Patients' PBMCs were immune‐phenotypically characterised *via* flow cytometry. Antibodies and procedures have been previously described.^19^, ^34^ For analyses of HDL‐treated HD T cells, cells were stained with fluorochrome‐conjugated antibodies according to manufacturers' instructions. A complete list of antibodies is provided in Supplementary table 5. Samples were then assessed on a Cytek NL‐3000 spectral flow cytometer (Cytek Biosciences, Fremont, CA, USA). The collected data were analysed using FlowJo V10 (FlowJo LLC, Ashland, OR, USA). Signal intensity was defined as the median fluorescence intensity (MFI).

### HDL treatment of T cells

HD‐derived T cells were isolated using the Pan T cell isolation kit (Miltenyi Biotec, Bergisch Gladbach, Germany). T cells of > 98% purity were activated with anti‐CD2/CD3/CD28 microbeads (Miltenyi Biotec) in the absence or presence of HDL (Sigma‐Aldrich, Saint Louis, MO, USA) for 120 h. Afterwards, cells were restimulated with PMA/ionomycin (Sigma‐Aldrich). BD GolgiPlug (BD Biosciences, Franklin Lakes, NJ, USA) was added simultaneously, and cells were incubated for an additional 4 h.

### Statistical analysis

Analysis was performed using R studio (posit, Boston, MA, USA)/R version 4.2.2^66^, ^67^, ^68^, ^69^, ^70^, ^71^, ^72^, ^73^, ^74^, ^75^, ^76^, ^77^ and GraphPad Prism Version 10 (GraphPad Software Inc., San Diego, CA, USA). Data were tested for outliers using the boxplot methods.^74^ After removing outliers, data were tested for normality and homogeneity. Multiple comparisons of patient‐derived data were performed using either a mixed‐effects model if there were missing values or a two‐way ANOVA with Bonferroni's multiple comparisons tests. For testing the impact of transplant‐related factors on HDL serum levels, a linear mixed‐effects model was used. HDL level was specified as the dependent variable; GvHD status (pre‐ vs. post‐onset), time point (days post‐transplant), sex, age, donor match (related vs. unrelated), conditioning regimen (myeloablative vs. non‐myeloablative), GvHD prophylaxis, GvHD grades, GvHD treatment (steroids), serum creatinine and CRP levels and occurrence of EBV, CMV, bacterial and fungal infections were included as fixed effects. A random intercept for each patient was included to model individual baseline HDL variability. Similarly, CD8^+^ T_EM_ frequencies were tested as the dependent variable with a random intercept for each patient and GvHD status, GvHD grade, time point, sex, age, donor match, day of engraftment, conditioning regimen, GvHD prophylaxis, ongoing GvHD treatment as well as infection/ virus reactivation at the respective time points. Model fitting was performed in R using the lme4 package,^78^ and the significance of fixed effects was assessed via the Satterthwaite approximation in the lmerTest package.^79^

One‐sample *t*‐tests were used to compare fold changes of HD T cells treated with HDL to activated T cells without HDL treatment (= untreated ctrl). For statistical analysis of *in vitro* proliferation and viability of HDL‐treated T cells, one‐way ANOVA with Bonferroni's multiple comparisons test was used.

## Author contributions

**Romy Böttcher‐Loschinski:** Conceptualization; investigation; writing – original draft. **Franziska Karl:** Formal analysis; investigation. **Diana Drettwan:** Formal analysis; investigation; writing – review and editing. **Johannes Wittmann:** Formal analysis; investigation; writing – review and editing. **Benedikt Jacobs:** Data curation. **Simon Völkl:** Data curation; resources; writing – review and editing. **Heiko Bruns:** Data curation; writing – review and editing. **Andreas Mackensen:** Resources. **Dimitrios Mougiakakos:** Conceptualization; data curation; supervision; writing – review and editing.

## Conflict of interest

The authors declare no conflict of interest.

## Supporting information


Supplementary figures 1–7.
**Supplementary tables 1–5**.

## Data Availability

The datasets generated during and/or analysed during the current study are available from the corresponding author upon reasonable request. The data are not publicly available because of privacy and/or ethical restrictions.
