# Supplementary material for: Elevated high‐density lipoprotein levels following acute graft‐versus‐host disease onset: a potential link to T‐cell dysfunction and increased relapse risk
Source: Clin Transl Immunology. 2025 Nov 3;14(11):e70060. doi: 10.1002/cti2.70060 (PMC12581179; doi:10.1002/cti2.70060)
Supplement: Supplementary file 1 — Supplementary figures 1–7. Supplementary tables 1–5. [file CTI2-14-e70060-s001.docx]

**
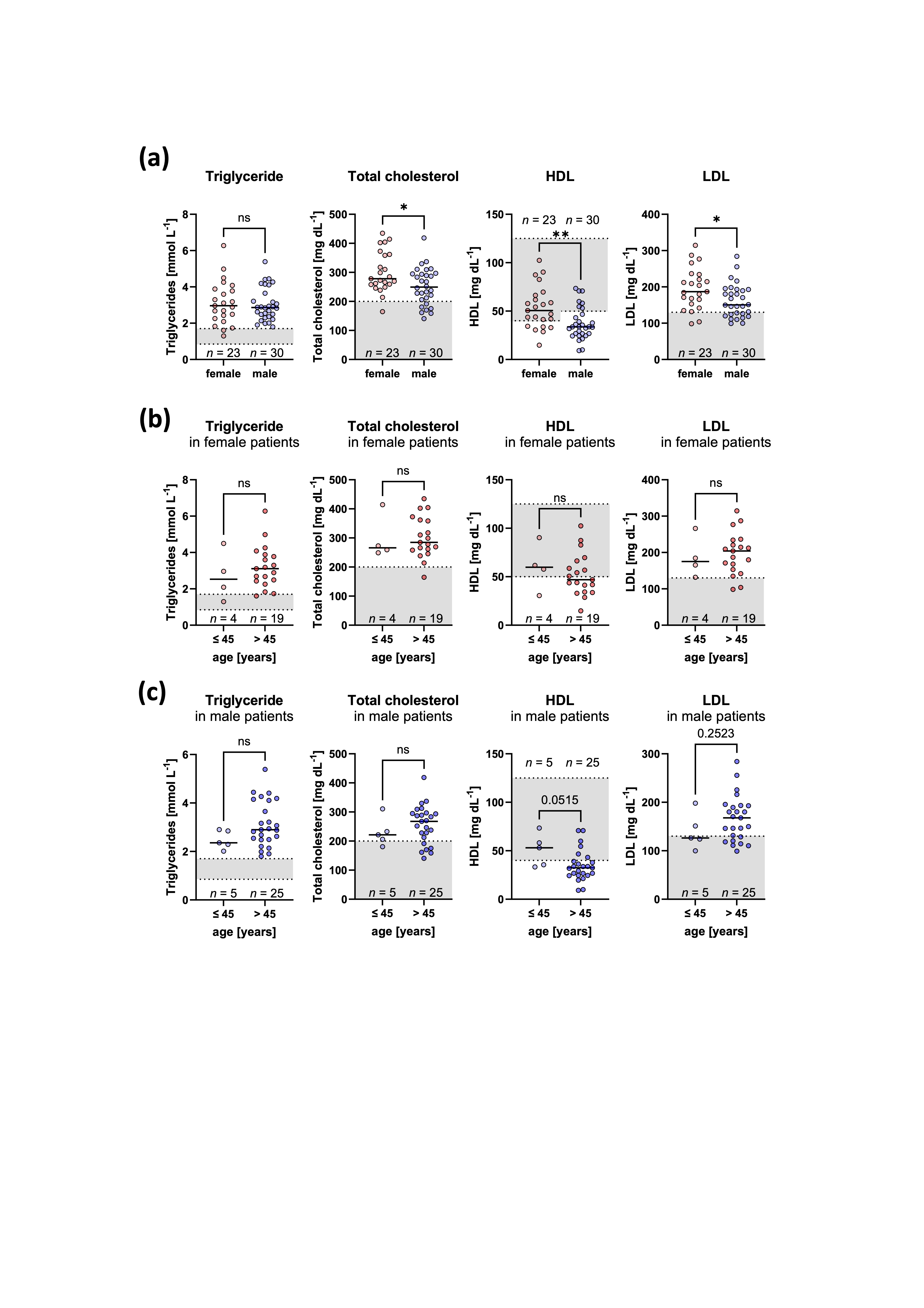
**

**Supplementary figure 1.** Serum analysis of allo-SCT patients. **(a)** Triglyceride, total cholesterol, HDL and LDL serum levels are presented as boxplots for female (*n* = 23) and male patients (*n* = 30). Gray boxes indicate healthy normal ranges. **(b)** Female and **(c)** male patients were grouped according their age into ≤ 45 years old (n = 4) and > 45 years (*n* = 19) old to compare serum triglyceride, total cholesterol, HDL, and LDL levels. Gray boxes indicate healthy normal ranges. HDL: high-density lipoprotein; LDL: low-density lipoprotein. Error bars indicate standard error of the mean. *P*-values: * < 0.05, ** < 0.01.

**
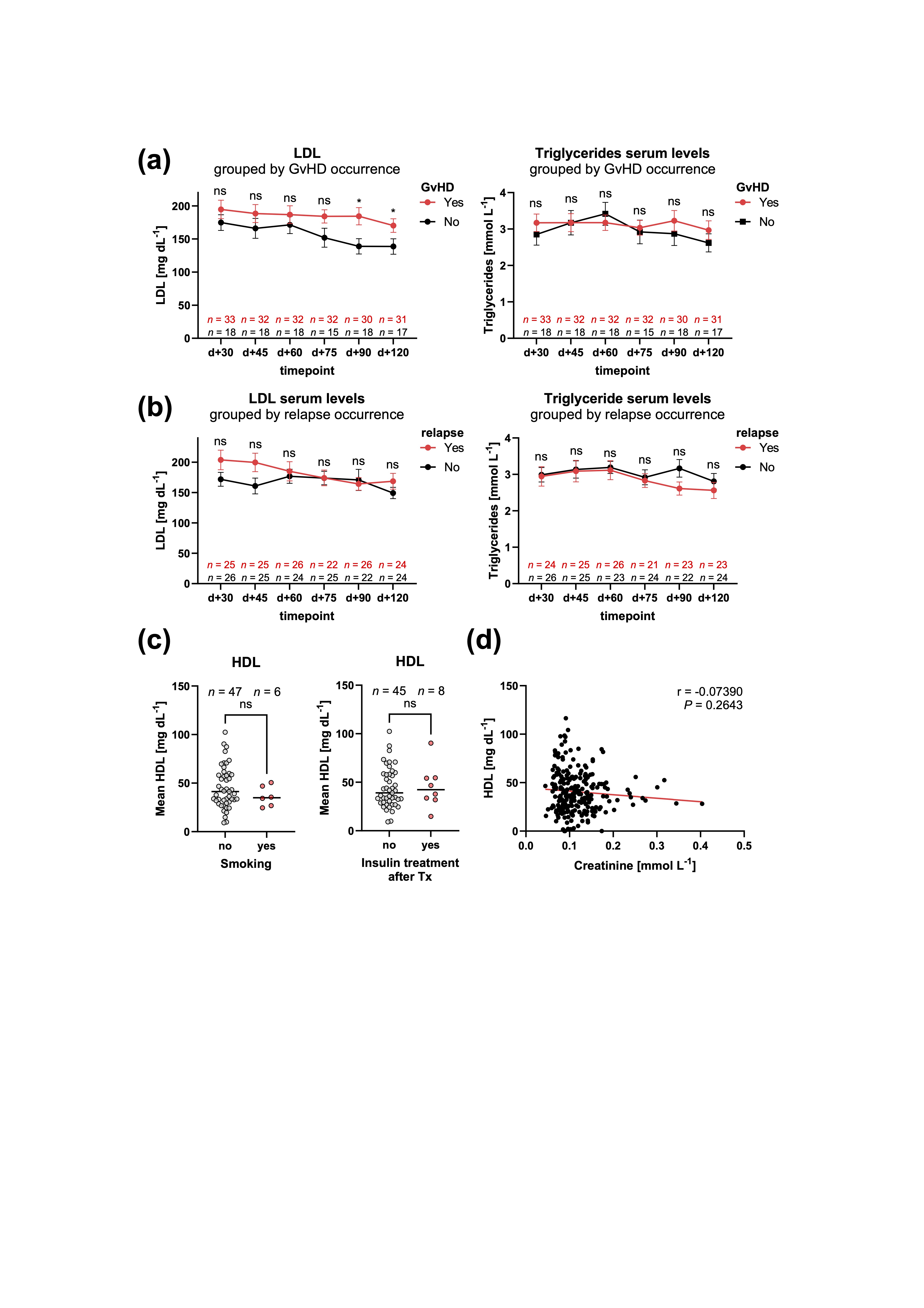
**

**Supplementary figure 2.** HDL serum levels and GvHD. Patients were grouped according to the occurrence of **(a)** aGvHD or **(b)** relapse. Changes in LDL and triglyceride serum levels over time are shown for these groups throughout the entire observation period. **(c)** Mean HDL serum level comparison between smoking and non-smoking patients, as well as between patients receiving insulin and those not receiving insulin after allo-SCT. **(d)** Correlation analysis (Pearson) of HDL serum levels and creatinine serum levels assessed at the same time point (n = 230). HDL: high-density lipoprotein; LDL: low-density lipoprotein; Tx: transplantation. Error bars indicate standard error of the mean. *P*-values: * < 0.05.

**
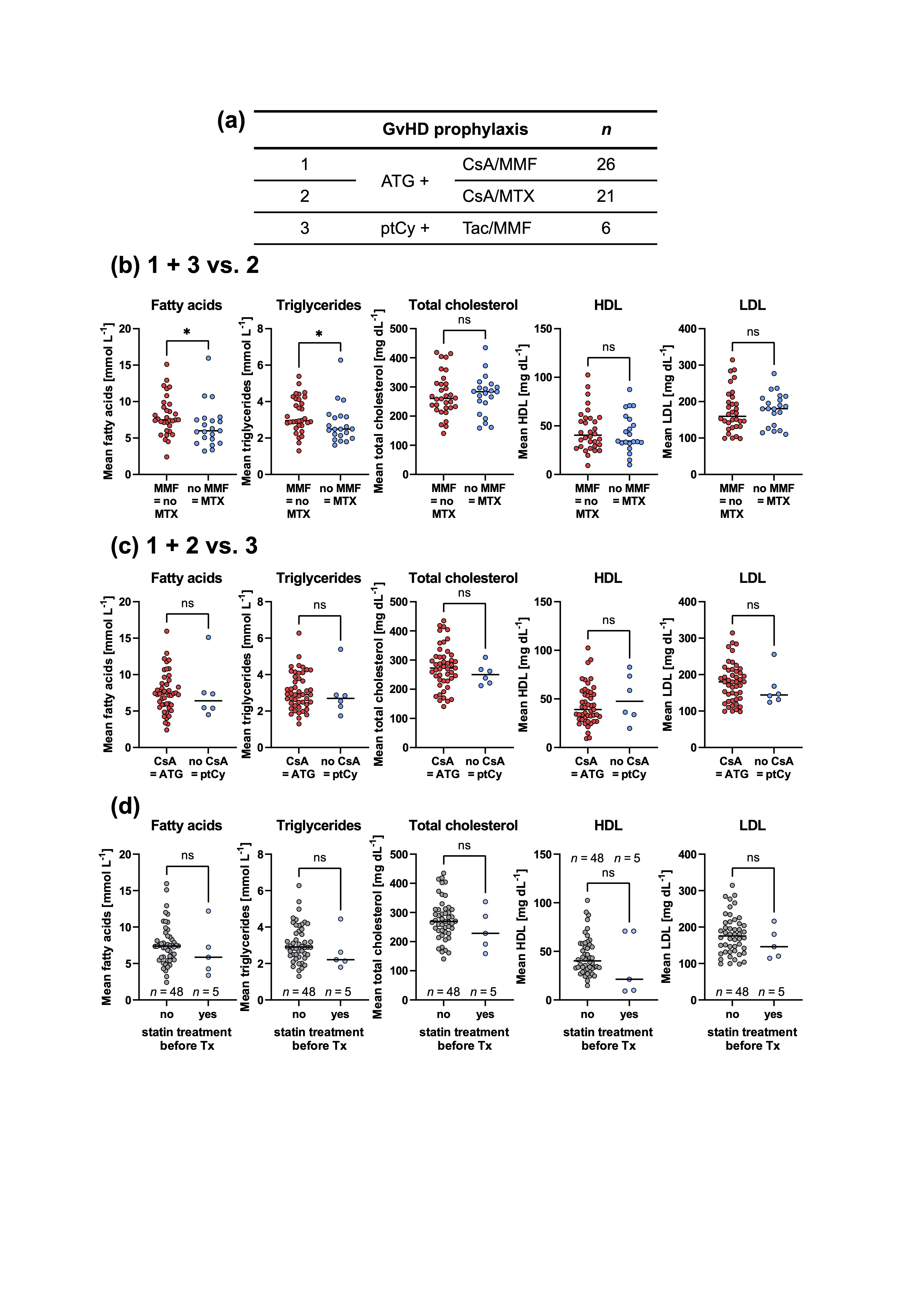
**

**Supplementary figure 3.** Serum levels of lipids and cholesterol in the context of GvHD prophylaxis and statin treatment before allo-SCT. **(a)** Overview of GvHD prophylaxis strategies. **(b)** Comparison of fatty acid, triglyceride, total cholesterol, HDL and LDL levels in patients receiving MMF as part of their GvHD prophylaxis and those not receiving MMF. **(c)** Comparison of fatty acid, triglyceride, total cholesterol, HDL and LDL levels in patients receiving CsA/ATG as part of their GvHD prophylaxis and those not receiving CsA, but ptCy. (d) Comparison of fatty acid, triglyceride, total cholesterol, HDL and LDL levels between patients who received statins prior to allo-SCT and patients with no statin treatment history. ATG: Anti-thymocyte globulin; CsA: Cyclosporine A; HDL: high-density lipoprotein; LDL: low-density lipoprotein; MMF: mycophenolate mofetil; MTX: methotrexate; ptCy: post-transplant cyclophosphamide; Tx: transplantation. *P*-values: * < 0.05.

**
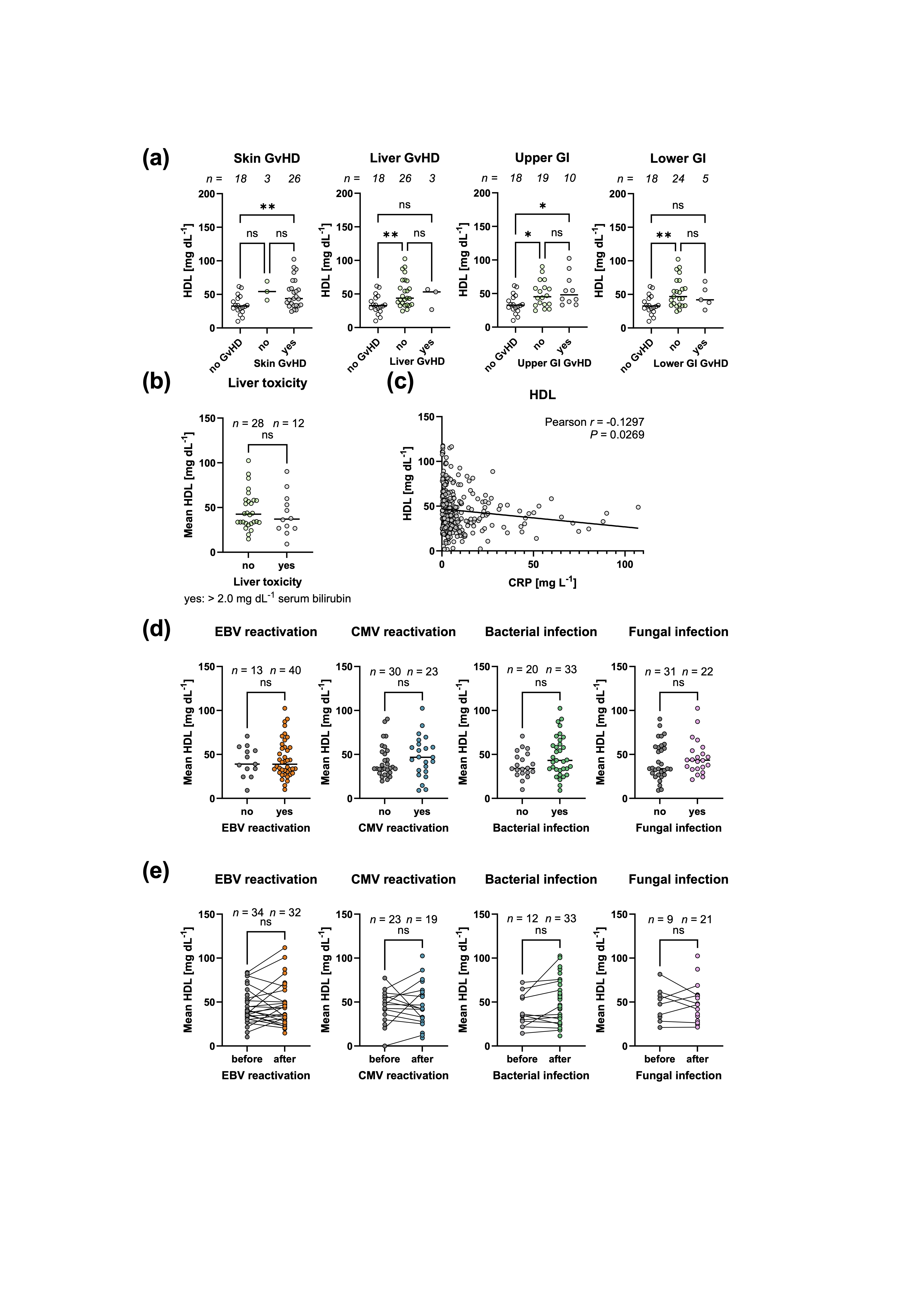
**

**Supplementary figure 4.** Impact of GvHD location and infections on HDL serum levels. **(a)** Mean HDL serum level are shown respective to the occurrence of skin, liver, lower gastrointestinal and/or upper gastrointestinal aGvHD. **(b)** Mean HDL serum level are shown respective to the occurrence of liver toxicity. The occurrence of liver toxicity was defined as > 2mg/dL serum bilirubin. **(c)** Correlation analysis (Pearson) of HDL serum levels and CRP serum levels assessed at the same time point (n = 291). **(d)** Mean HDL serum level comparison between patients experiencing EBV/CMV reactivation, bacterial or fungal infection and those who not. **(e)** Mean HDL serum level comparison of mean HDL serum levels before and after reactivation or infection onset. HDL: high-density lipoprotein. *P*-values: * < 0.05.

**
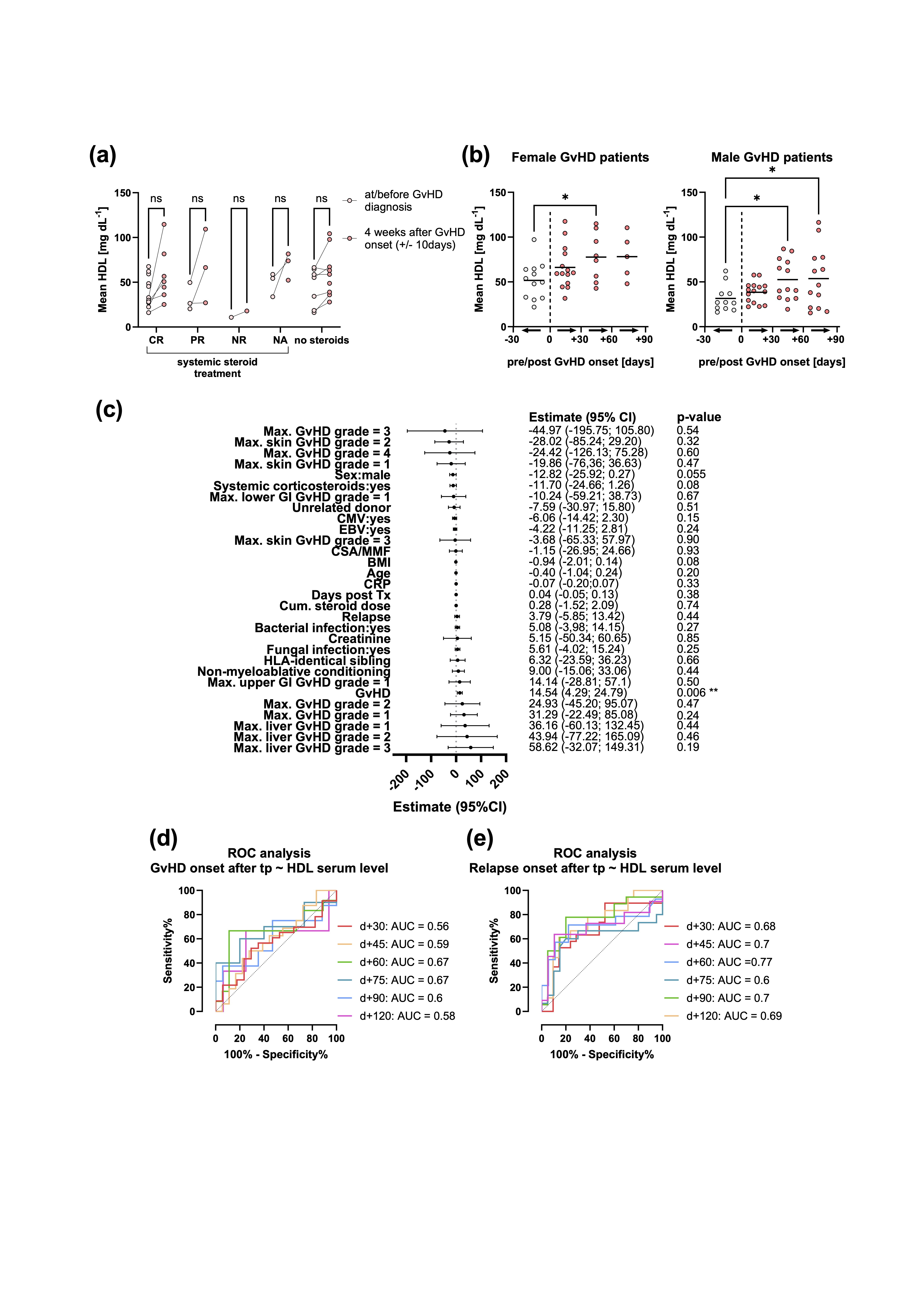
**

**Supplementary figure 5.** Impact of transplant-related factors on HDL serum levels. **(a)** A comparison of the mean HDL serum levels at/before GvHD onset and the four-week follow-up for patients treated with systemic corticosteroids, grouped by treatment status. **(b)** Mean HDL serum levels of female and male patients before and after GvHD onset, binned into 30-day intervals from 30 days before to 90 days after GvHD onset. **(c)** Forest plot of a linear mixed-effects model testing the impact of transplant-related factors on HDL serum levels. Error bars indicate CI. **(d)** ROC analysis of pre-GvHD HDL serum levels at six post-transplant timepoints by comparing the HDL levels from patients who never developed acute GvHD with those who developed GvHD after the respective sampling time. **(e)** ROC analysis of pre-relapse HDL serum levels at six post-transplant timepoints by comparing the HDL levels from patients who never relapsed and patients who relapsed after the respective sampling time. CR: complete remission; HDL: high-density lipoprotein; NA: not applicable; NR: no response; PR: partial remission. *P*-values: * < 0.05, ** < 0.01.

**
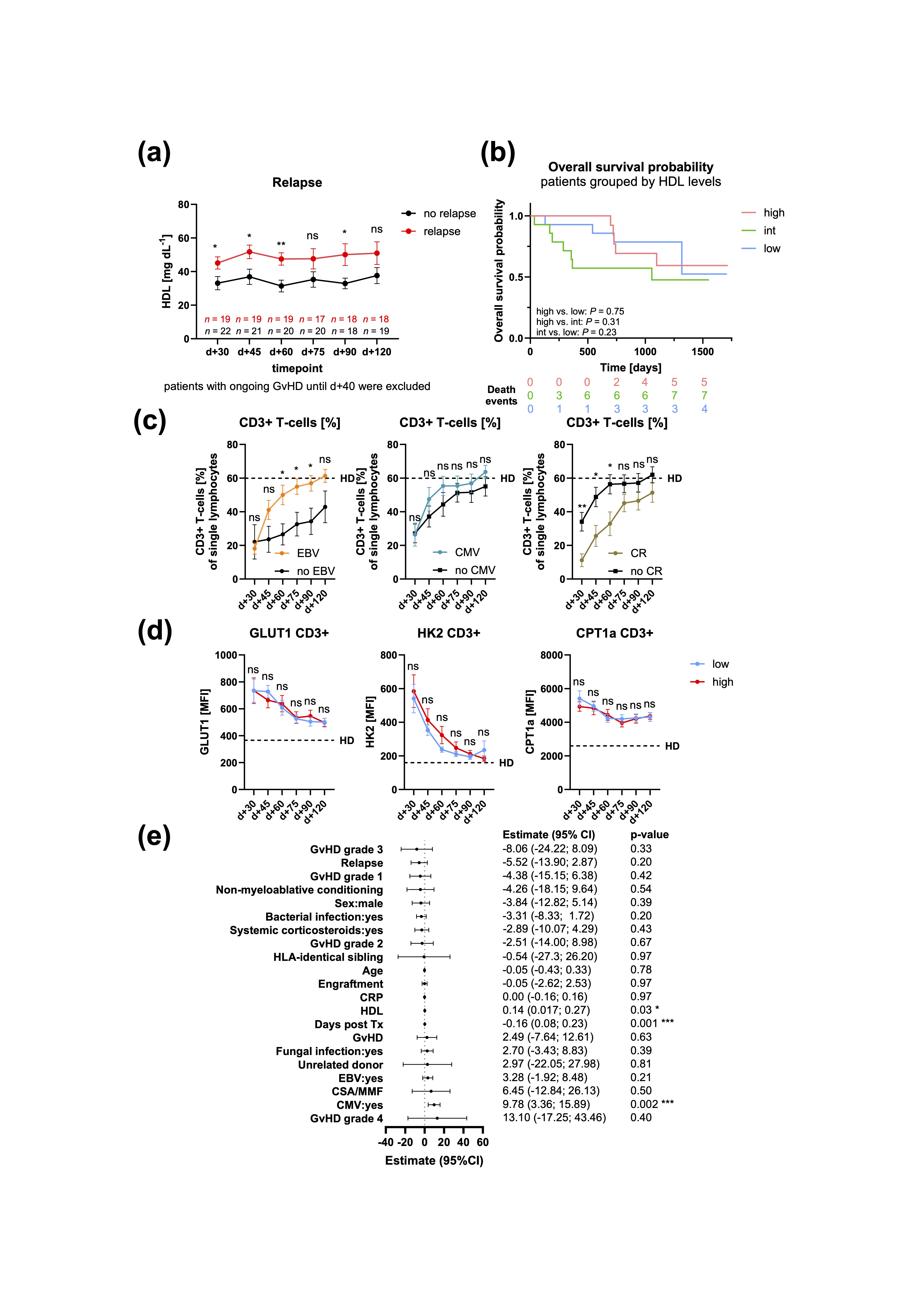
**

**Supplementary figure 6.** HDL extreme groups of allo-SCT patients. **(a)** Patients, except those who experienced GvHD before d+40 after allo-SCT, were grouped according to the occurrence of relapse. Changes in HDL serum levels over time are shown for these groups throughout the entire observation period. Error bars indicate SEM. **(b)** Survival probability of all three HDL groups. **(c)** The CD3+ T-cell frequency is shown for the entire observation period (n=11-30 per group and time point) for EBV/no EBV reaction, CMV/no CMV reactivation and no CR/no CR before allo-SCT. Error bars indicate SEM. **(d)** The MFIs of GLUT1, HK2 and CPT1a of total T-cells are shown for the entire observation period (n=9-14 per group and time point). Error bars indicate SEM. **(e)** Forest plot of a linear mixed-effects model testing the impact of transplant-related factors on CD8^+^ T_EM_ frequencies of allo-SCT patients. Error bars indicate CI. MFI: median fluorescence intensity. *P*-values: * < 0.05, ** < 0.01, *** < 0.001.

**
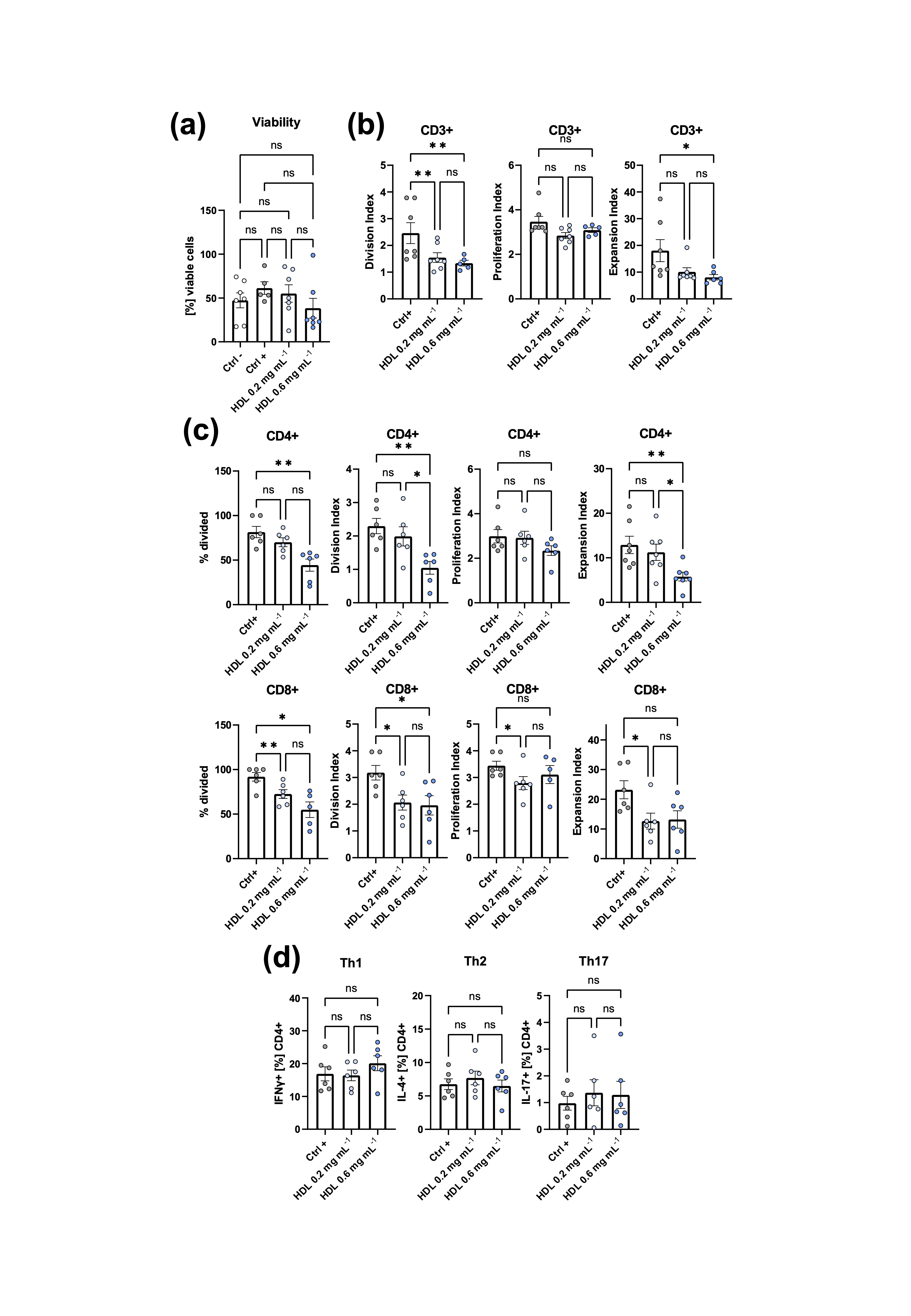
**

**Supplementary figure 7.** *In vitro* HDL treatment of HD T cells. **(a)** Viability of T cells after cell culture with/without HDL (*n* = 6). **(b, c)** Proliferation statistics of CD3+ **(b)** and CD4+/CD8+ T cells **(c)** after 120h of cell culture with/without HDL (*n* = 5). **(d)** Frequencies of either IFNγ^+^, IL-4^+^ or IL-17^+^ T cells are shown after 120h of cell culture with/without HDL (*n* = 6). Error bars indicate SEM. HDL: high-density lipoprotein. *P*-values: * < 0.05, ** < 0.01.

**Supplementary table 1 Appendix to Table 1: Descriptive statistics of HDL extreme groups.**

| **Variable** | **High**  **(*n* = 13)** | **Int**  **(*n* = 14)** | **Low**  **(*n* = 14)** | ***P* overall** |
| --- | --- | --- | --- | --- |
| **Diagnosis before Tx:** |  |  |  | 0.852 |
| AML/MDS | 7 (53.8%) | 9 (64.3%) | 8 (57.1%) |  |
| other | 6 (46.2%) | 5 (35.7%) | 6 (42.9%) |  |
| **Engraftment [days]:** | 14.0 [9.00;16.0] | 17.0 [9.00;23.0] | 14.0 [9.00;21.0] | 0.065 |
| **GvHD prophylaxis:** |  |  |  | 0.394 |
| CSA/MMF | 6 (46.2%) | 9 (64.3%) | 7 (50.0%) |  |
| CSA/MTX | 5 (38.5%) | 3 (21.4%) | 7 (50.0%) |  |
| cylcophosphamide/ tacrolimus/MMF | 2 (15.4%) | 2 (14.3%) | 0 (0.00%) |  |
| **Conditioning:** |  |  |  | 0.152 |
| myeloablative | 6 (46.2%) | 10 (71.4%) | 5 (35.7%) |  |
| non-myeloablative | 7 (53.8%) | 4 (28.6%) | 9 (64.3%) |  |
| **Maximal overall aGvHD grade:** |  |  |  | 0.34 |
| 1 | 6 (66.7%) | 6 (85.7%) | 2 (28.6%) |  |
| 2 | 2 (22.2%) | 1 (14.3%) | 2 (28.6%) |  |
| 3 | 0 (0.00%) | 0 (0.00%) | 2 (28.6%) |  |
| 4 | 1 (11.1%) | 0 (0.00%) | 1 (14.3%) |  |
| **Skin GvHD:** |  |  |  | 0.798 |
| No | 6 (50.0%) | 6 (50.0%) | 8 (61.5%) |  |
| Yes | 6 (50.0%) | 6 (50.0%) | 5 (38.5%) |  |
| **Liver GvHD:** |  |  |  | 1.000 |
| No | 12 (100%) | 12 (100%) | 12 (92.3%) |  |
| Yes | 0 (0.00%) | (0.00%) | 1 (7.69%) |  |
| **Upper GI GvHD:** |  |  |  | 0.854 |
| No | 10 (83.3%) | 11 (91.7%) | 10 (76.9%) |  |
| Yes | 2 (16.7%) | 1 (8.33%) | 3 (23.1%) |  |
| **Lower GI GvHD:** |  |  |  | 1.000 |
| No | 11 (91.7%) | 11 (91.7%) | 11 (84.6%) |  |
| Yes | 1 (8.3%) | 1 (8.3%) | 2 (15.4%) |  |
| **Steroids:** |  |  |  | 0.919 |
| No | 8 (61.5%) | 10 (71.4%) | 9 (64.3%) |  |
| Yes | 5 (38.5%) | 4 (28.6%) | 5 (35.7%) |  |
| **Day 28 Cumulated Steroid Dose [mg kg^-1^]** | 12.03  [2.26; 33.1] | 12.07  [9.18; 17.9] | 13.2  [5.11; 32.1] | 0.984 |

AML acute myeloid leukemia, MDS myelodysplastic syndrome, CSA cyclosporine A, MMF mycophenolate mofetil, MTX methotrexate, Tx transplantation

**Supplementary table 2 Multivariate analysis of T-cell frequency normalization.**

| **Time to normalization of T-cell frequency [days]** | | **HR (univariable)** | **HR (multivariable)** |
| --- | --- | --- | --- |
| Sex | | | |
| female | 23 (43.4) | - | - |
| male | 30 (56.6) | 1.01  (0.52-1.98, *P* = 0.971) | 0.65  (0.21-2.04, *P* = 0.462) |
| Age at Tx | | | |
| Mean (SD) | 54.7 (13.6) | 0.99  (0.97-1.01, *P* = 0.208) | 0.99  (0.96-1.02, *P* = 0.563) |
| Donor | | | |
| haploidentical family  member | 6 (11.3) | - | - |
| HLA-identical sibling | 8 (15.1) | 1.14  (0.25-5.08, *P* = 0.868) | 3.94  (0.39-39.30, *P* = 0.243) |
| unrelated donor | 39 (73.6) | 1.64  (0.50-5.43, *P* = 0.416) | 1.62  (0.21-12.44, *P* = 0.641) |
| Conditioning | | | |
| myeloablative | 24 (45.3) | - | - |
| non-myeloablative | 29 (54.7) | 1.25  (0.64-2.44, *P* = 0.515) | 3.55  (0.46-27.38, *P* = 0.223) |
| CR status at Tx | | | |
| No | 34 (64.2) | - | - |
| Yes | 19 (35.8) | 0.37  (0.17-0.80, *P* = 0.011) | 0.26  (0.09-0.77, *P* = 0.015) |
| Steroids | | | |
| No | 29 (54.7) | - | - |
| Yes | 24 (45.3) | 1.00  (0.51-1.96, *P* = 0.993) | 0.57  (0.22-1.45, *P* = 0.237) |
| GvHD prophylaxis | | | |
| CSA/MMF | 26 (49.1) | - | - |
| CSA/MTX | 21 (39.6) | 1.30  (0.65-2.61, *P* = 0.462) | 0.36  (0.05-2.79, *P* = 0.325) |
| cylcophosphamide/  tacrolimus/MMF | 6 (11.3) | 0.72  (0.21-2.46, *P* = 0.602) | NA  (NA-NA, *P* = NA) |
| Diagnosis | | | |
| AML/MDS | 28 (52.8) | - | - |
| other | 25 (47.2) | 1.57  (0.81-3.06, *P* = 0.181) | 2.21  (0.89-5.49, *P* = 0.086) |

**Appendix to Supplementary table 2 Multivariate analysis of T-cell frequency normalization.**

| **Time to normalization of T-cell frequency [days]** | | **HR (univariable)** | **HR (multivariable)** |
| --- | --- | --- | --- |
| EBV reactivation | | | |
| no | 13 (24.5) | - | - |
| yes | 40 (75.5) | 2.61  (1.00-6.80, P = 0.049) | 6.47  (1.23-34.03, P = 0.027) |
| CMV reactivation | | | |
| no | 30 (56.6) | - | - |
| yes | 23 (43.4) | 1.81  (0.93-3.53, *P* = 0.080) | 1.06  (0.43-2.58, *P* = 0.901) |
| HDL group | | | |
| high | 13 (31.7) | - | - |
| int | 14 (34.1) | 0.97  (0.37-2.53, *P* = 0.955) | 2.34  (0.51-10.72, *P* = 0.274) |
| low | 14 (34.1) | 3.03  (1.19-7.76, *P* =0.020) | 5.47  (1.53-19.55, *P* = 0.009) |

GvHD acute graft-versus-host disease, AML acute myeloid leukemia, CMV Cytomegalovirus, CR complete remission, CSA cyclosporine A, EBV Epstein–Barr virus, HR Hazard ratio, MDS myelodysplastic syndrome, MMF mycophenolate mofetil, MTX methotrexate, PR partial remission, SD stable disease, Tx transplantation

**Supplementary table 3 Appendix to Table 2: alloSCT patient characteristics.**

| **Variable** | **Patients (*n* = 53)** |
| --- | --- |
| **Statin treatment before allo-SCT:** |  |
| Yes | 5 (9.4%) |
| No | 48 (90.6%) |
| **Smoking:** |  |
| Yes | 6 (11.3%) |
| No | 47 (88.7%) |
| **Diabetes/insulin treatment after Tx:** |  |
| Yes | 8 (15.1%) |
| No | 45 (84.9%) |
| **Parenteral nutrition:** | 53 (100%) |
| **CMV mismatch:** |  |
| R0D0 | 19 (35.8%) |
| R1D1 | 20 (37.7%) |
| R0D1 | 3 (5.7%) |
| R1D0 | 11 (20.8%) |

**Supplementary table 4 Sample quantity per timepoint included.**

| **Time point post transplantation [days]** | ***n*** |
| --- | --- |
| d+30 | 51 |
| d+45 | 50 |
| d+60 | 50 |
| d+70 | 47 |
| d+90 | 49 |
| d+120 | 49 |

**Supplementary table 5 Flow cytometry antibodies and dyes.**

| Antigen | Fluorochrome | Clone | Isotype | Identifier | Company |
| --- | --- | --- | --- | --- | --- |
| CD3 | BV786 | Okt 03 | Mouse IgG2a, κ | 566781 | BD Biosciences, Franklin Lakes, NJ, USA |
| CD8 | AF488 | RPA-T8 | Mouse IgG1, κ | 557704 |  |
| CD4 | PE/Fire640 | SK3 | Mouse IgG1, κ | 344664 | Biolegend, San Diego, CA, USA |
| CD4 | PE/Cy7 | SK3 | Mouse IgG1, κ | 344612 |  |
| CD8 | BV570 | RPA-T8 | Mouse IgG1, κ | 301038 |  |
| CD25 | BV510 | M-A251 | Mouse IgG1, κ | 356120 |  |
| CD45RO | PE/Cy7 | UCHL1 | Mouse IgG2a, κ | 304230 |  |
| CD69 | BV650 | FN50 | Mouse IgG1, κ | 310934 |  |
| CD127 | BV711 | A019D5 | Mouse IgG1, κ | 351328 |  |
| CD137 | BV750 | 4B4-1 | Mouse IgG1, κ | 309844 |  |
| CD197/CCR7 | APC/Fire810 | G043H7 | Mouse IgG2a, κ | 353264 |  |
| CD279/PD-1 | BV421 | EH12.2H7 | Mouse IgG1, κ | 329920 |  |
| Granzyme B | Pacific Blue | GB11 | Mouse IgG1, κ | 515408 |  |
| IFN-γ | BV750 | 4S.B3 | Mouse IgG1, κ | 502550 |  |
| IL-2 | AF647 | JES6-5H4 | Rat IgG2b, κ | 503814 |  |
| KLRG1 | PerCP-Cy5.5 | SA231A2 | Mouse IgG2a, κ | 367708 |  |
| Perforin | PE-Dazzle594 | dG9 | Mouse IgG2b, κ | 308132 |  |
| Proliferation | VPD450 |  |  | 562158 | BD Biosciences, Franklin Lakes, NJ, USA |
| TNFα | PE/Cy7 | MAb11 | Mouse IgG1, κ | 502930 | Biolegend, San Diego, CA, USA |
| TOX | VioB515 | REA473 | recombinant human IgG1 | 130-129-208 | Miltenyi, Bergisch Gladbach, Germany |
| Viability | Zombie NIR |  |  | 423106 | Biolegend, San Diego, CA, USA |
